# Access-Related Hand Dysfunction After Hemodialysis Access Placement

**DOI:** 10.1016/j.ekir.2025.103765

**Published:** 2026-01-02

**Authors:** Kyoungrae Kim, Trace Thome, Lauren Stone, Nicholas Vugman, Eric M. Kunz, Samuel Alvarez, Qingping Yang, Kerri A. O’Malley, Erik M. Anderson, Brian Fazzone, Pavel Mazirka, Jesseca Antoine, Scott A. Berceli, Terence E. Ryan, Salvatore T. Scali

**Affiliations:** 1 Department of Applied Physiology and Kinesiology, University of Florida, Gainesville, Florida, USA; 2 Division of Vascular Surgery and Endovascular Therapy, Department of Surgery, University of Florida, Gainesville, Florida, USA; 3 Division of Vascular Surgery, Department of Surgery, Malcolm Randall Veterans Affairs Medical Center, Gainesville, Florida, USA; 4 Department of Applied Physiology and Kinesiology, Center for Exercise Science, University of Florida, Gainesville, Florida, USA; 5 Department of Applied Physiology and Kinesiology, Myology Institute, University of Florida, Gainesville, Florida, USA

**Keywords:** access failure, chronic kidney disease, end-stage kidney disease, hand dysfunction, hemodialysis access, muscle atrophy

## Abstract

**Introduction:**

Patients with chronic kidney disease (CKD) or end-stage kidney disease (ESKD) frequently experience access-related hand dysfunction (ARHD) following hemodialysis access placement, negatively impacting clinical outcomes and quality of life. Despite its prevalence, the mechanisms underlying ARHD remain poorly understood, and its impact on subsequent access maturation failure has not been well-characterized.

**Methods:**

We conducted a longitudinal study of 39 patients with CKD or ESKD undergoing hemodialysis access surgery. Functional assessments, including grip strength, finger pressure, patient-reported hand or arm disability, dexterity, and sensory testing were performed preoperatively and 6 weeks postoperatively. Brachioradialis muscle biopsies were analyzed for morphological changes, mitochondrial function, and transcriptomic profiles. Dialysis access maturation failure was evaluated at 6-month follow-up.

**Results:**

Six weeks after surgery, grip strength declined by 13.5 ± 19.4% (*P* = 0.0001), finger pressure decreased by 30.6 ± 38.0 mm Hg (*P* = 0.0008), and patient-reported limb disability scores worsened (*P* = 0.0213). Dexterity and sensation showed no changes. Histological analysis revealed an approximately 12% reduction in myofiber cross-sectional area (*P* = 0.0340), which is significantly correlated with grip strength (*P* = 0.0488). Mitochondrial content increased (*P* = 0.0683) and was inversely correlated with finger pressure (*P* = 0.0321), whereas mitochondrial respiration and antioxidant capacity remained unchanged. RNA sequencing revealed alterations in the genes regulating myofiber development. Notably, patients with ARHD had an approximately 20% higher incidence of unassisted maturation failure at 6 months (*P* = 0.0046).

**Conclusion:**

In patients with CKD or ESKD, hemodialysis access surgery results in ARHD, which is associated with muscle atrophy, altered transcriptomic profiles, and higher incidence of access maturation failure. These findings underscore the need for early identification and targeted interventions to prevent ARHD and improve dialysis-access surgery outcomes.

## Graphical abstract

**Figure:**
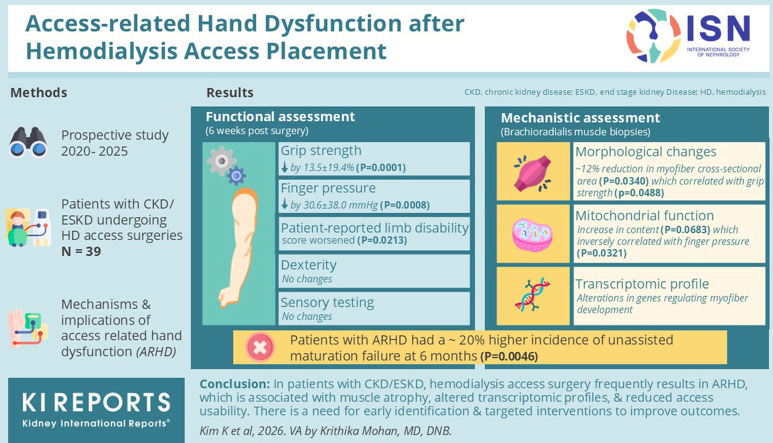

Currently, > 450,000 patients with advanced CKD or ESKD in the United States are receiving chronic in-center hemodialysis.^1^ The success of this life-sustaining therapy depends on the establishment of a durable and well-functioning vascular access,^2^^,^^3^ with arteriovenous fistula (AVF) or arteriovenous graft (AVG) representing the most commonly employed modalities. Although the primary clinical focus is centered on ensuring vascular access patency and functionality, ≤ 60% of patients experience ARHD following vascular access placement.^4^ The clinical spectrum of ARHD ranges from subtle impairments in sensation and coordination to paraparesis and digital gangrene in cases of severe hand ischemia (i.e., “steal syndrome”).^5^, ^6^, ^7^, ^8^, ^9^ With these manifestations, patients undergoing hemodialysis access surgery often experience reduced grip strength and greater perceived limb disabilities, which are associated with increased risks of morbidity and mortality, as well as poor quality.^10^, ^11^, ^12^, ^13^ Moreover, unresolved ARHD can negatively affect both dialysis access longevity and treatment adherence, increasing the risk of poor health outcomes.^14^, ^15^, ^16^

Importantly, these functional impairments may compromise the clinical usability of the access itself. Difficulty using the limb because of weakness or pain may reduce a patient’s tolerance of cannulation, interfere with physical therapy or rehabilitation efforts that support access maturation and hemodynamic adaptation, or ultimately lead to abandonment of an otherwise technically functional access. However, the relationship between early postoperative ARHD and longer-term access outcomes such as unassisted maturation failure has not been well-defined.

Historically, hemodynamic disturbances such as “steal syndrome” following the placement of AVF or AVG have been proposed as the predominant cause of ARHD.^17^^,^^18^ However, steal syndrome leading to surgical remediation occurs in only 5% to 7% of patients who undergo dialysis-access surgery.^19^^,^^20^ In contrast, previous studies have documented decreases in grip strength and increased reports of hand or arm disability even in the absence of overt “steal syndrome.”^4^^,^^21^ Furthermore, the weak correlation between hemodynamic alterations and the severity of observed hand and arm disabilities suggests that other factors contribute to the development of ARHD.^4^^,^^22^^,^^23^ Despite this, the underlying pathophysiological mechanisms responsible for this debilitating condition remains largely unexplored, creating a critical gap in understanding how biological and mechanical factors, beyond hemodynamic changes, drive development of postoperative ARHD. Because ischemia alone cannot fully explain ARHD, we hypothesized that structural and bioenergetic alterations in skeletal muscle contribute to functional deficit.

In this study, we conducted a prospective investigation in patients with CKD or ESKD undergoing dialysis access surgery to explore mechanisms contributing to ARHD beyond hemodynamic disturbances. Hand and arm function were assessed preoperatively and at 6 weeks postoperatively, using a comprehensive battery of functional tests. In parallel, forearm muscle biopsies were obtained to evaluate changes in muscle morphology, mitochondrial function, and transcriptomic profiles, providing mechanistic insights into how cellular and molecular changes underlie observed functional impairments. In addition, we evaluated whether early ARHD correlates with vascular access maturation at 6 months, aiming to clarify its role as a clinically relevant and potentially modifiable determinant of access success.

## Methods

### Study Design

**Figure 1:**
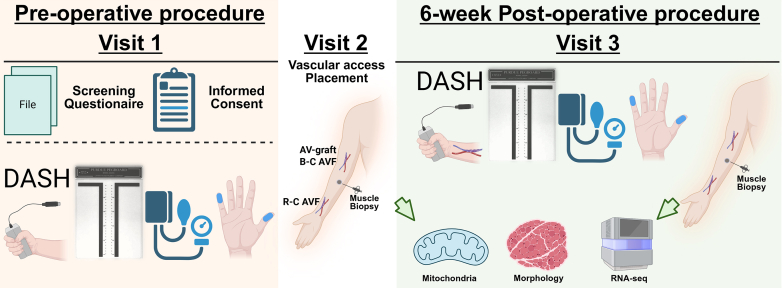
Schematic overview of the experimental procedure. BC-AVF, brachiocephalic arteriovenous fistula; RC-AVF, radiocephalic arteriovenous fistula; DASH, Disability of Arm, Shoulder and Hand.

A cohort of 39 patients diagnosed with CKD or ESKD was recruited between 2020 and 2025 from 2 clinical centers: University of Florida Health and Malcom Randall Veterans Affairs Medical Center (Figure 1). At the initial visit, after completing the screening questionnaire and providing informed consent, patients underwent preoperative assessments to evaluate baseline hand function in both extremities. These evaluations included grip strength measurement; as well as hemodynamic measurement including hand and wrist systolic blood pressure, Disability of the Arm, Shoulder, and Hand (DASH) questionnaire, pegboard dexterity assessment, and monofilament sensory test. Following these assessments, patients were scheduled for the hemodialysis access creation via AVF or AVG. Detailed demographic information and surgical details were recorded in an electronic data capture system. During the second visit, under local or general anesthesia, a preoperative muscle biopsy was obtained from the brachioradialis muscle to assess changes in muscle morphology, mitochondrial function, and transcriptomic profiles. Six weeks after vascular access placement, patients returned to the surgery clinic for a follow-up evaluation. During this visit, postoperative functional assessments were conducted, replicating the preoperative evaluations, and a postoperative muscle biopsy was performed under local anesthesia. Grip strength was specified as the primary functional outcome *a priori*; all other assessments were considered secondary or exploratory end points. Routine follow-up assessments were conducted over a 6-month period to monitor hemodialysis access maturation and/or the usability of cannulation. The detailed methods are provided in the Supplementary Methods.

### Dialysis Access Maturation and Failure Definitions

Vascular access maturation was assessed at routine postoperative follow-up (typically between 4–6 weeks postoperatively when clinical decisions to “release the access for use” were made), with formal evaluation at 6 months following access creation. Maturation was defined as successful functional usability of the access, operationalized by the ability to support repeated (e.g., > 3 consecutive) 2-needle cannulation sessions for hemodialysis with adequate flow, consistent with standard clinical practice rather that solely anatomic criteria (e.g., rule of 6s).

Unassisted maturation failure was defined as failure of the access to achieve functional usability without the need for endovascular or surgical intervention. Assisted maturation failure was defined as failure to achieve functional usability despite ≥ 1 assistive interventions (e.g., angioplasty or surgical revision). Access failure was defined as loss of access patency resulting in abandonment of the access. Analyses focused on primary access outcomes during the 6-month follow-up period.

### Statistical Analysis

Normality of the data was assessed using Shapiro–Wilk test and visual inspection of QQ plots. For hand and arm functional measurements, paired *t* tests were used to compare outcomes between the access and nonaccess limbs, as well as between pre- and postoperative conditions. Muscle histopathological evaluations, which considered variability in fiber counts, were analyzed using ratio paired *t* tests to ensure accurate quantification of changes. A mixed-effects model was applied for repeated measures data, and Šídák's *post hoc* test was conducted for multiple comparisons when significant interactions were detected. To assess relationships between 2 continuous variables, Pearson correlation analysis was employed. Multivariable linear models were employed to evaluate the impact of ethnicity, dialysis status, and hemodialysis access configuration as covariates on ARHD. Sensitivity models incorporating baseline grip strength, body mass index, and dialysis dependence as covariates confirmed persistence of the observed effect. For bulk RNA sequencing data, the Benjamini–Hochberg method was used to calculate false discovery rate–corrected *P-*values. To compare the proportions of vascular access failure between patients with and without ARHD, a Chi-square test was used via VassarStats. All statistical analyses were performed using R-Studio (R Foundation for Statistical Computing, Vienna, Austria) and GraphPad Prism (v.9.0; GraphPad, La Jolla, CA), with a *P*-value < 0.05 considered statistically significant. Data are presented as means ± SD unless otherwise stated.

## Results

### Patients Characteristics

Demographics, comorbidities, and access configurations of the patients are described in Table 1. The cohort was predominantly male (87.2%) and included 59.0% Caucasians and 35.9% African Americans. The 3 most prevalent comorbidities among study patients were hypertension (94.9%), diabetes (53.8%), and hyperlipidemia (66.7%). At the time of surgery, 56.4% of patients were receiving chronic hemodialysis using a tunneled dialysis catheter. Hemodialysis access configurations varied, with 46.2% of patients receiving a brachiocephalic AVF (BC-AVF), 43.6% with a radiocephalic AVF (RC-AVF), and 10.3% with an AVG.

**Table 1:** Demographics, comorbidities, and access outcomes (*N* = 39)

Variable	*n* (%)
Age, mean ± SD, yr	66.3 ± 13.2
Male	34 (87.2)
BMI, mean ± SD, kg/m^2^	25.7 ± 8.9
Race	
White/Caucasian	23 (59.0)
Black/African American	14 (35.9)
Other	2 (5.1)
Comorbidities	
Hypertension	37 (94.9)
Diabetes	21 (53.8)
Hyperlipidemia	26 (66.7)
Congestive heart failure	8 (20.5)
Medication used	
Aspirin	13 (33.3)
ACE inhibitors	10 (25.6)
Angiotensin receptor blocker	12 (30.8)
Beta blocker or calcium channel blocker	36 (92.3)
Statin	25 (64.1)
Etiology of renal disease	
Diabetic nephropathy	12 (30.8)
Hypertensive nephropathy	13 (33.3)
Polycystic kidney disease	2 (5.1)
Other diagnosis	12 (30.8)
Duration of hemodialysis	
Not on hemodialysis	17 (43.6)
< 6 mos	4 (10.3)
6–12 mos	13 (33.3)
> 12 mos	5 (12.8)
Prior AV access surgery history	
None	34 (87.2)
Access configuration	
Brachial-cephalic AVF	18 (46.2)
Radial-cephalic AVF	17 (43.6)
AVF Graft	4 (10.3)
Access on dominant limb side	11 (28.2)
Previous vascular access attempt	5 (12.8)

ACE, angiotensin-converting enzyme; AV, arteriovenous; AVF, arteriovenous fistula BMI, body mass index.

### ARHD is Prevalent Following Hemodialysis Access Placement

**Figure 2:**
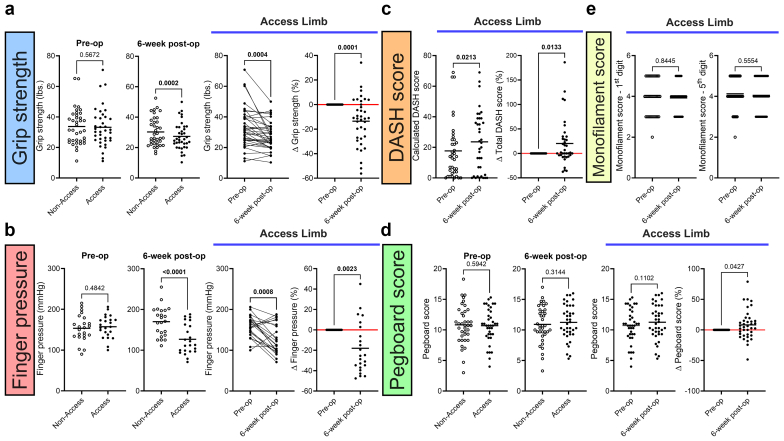
Access-related hand dysfunction emerges as a distinctive manifestation in the early phase of postoperative surgery. (a) Grip strength comparison between nonaccess and access limbs at pre-op and 6 weeks post-op (left 2 panels) and comparison of raw values and percentage delta changes in grip strength for the access limb from pre-op to 6 weeks post-op (right 2 panels). (b) Finger pressure comparison between nonaccess and access limbs at pre-op and 6 weeks post-op (left 2 panels) and comparison of raw values and percentage delta changes in finger pressure for the access limb from pre-op to 6 weeks post-op (right 2 panels). (c) Comparison of calculated DASH scores (left panel) and percentage delta changes in total DASH scores (right panel) between pre-op and 6 weeks post-op. (d) Pegboard score comparison between nonaccess and access limbs at pre-op and 6 weeks post-op (left 2 panels) and comparison of raw values and percentage delta changes in pegboard scores for the access limb from pre-op to 6 weeks post-op (right 2 panels). (e) Comparison of monofilament scores for the first (left) and fifth (right) digits between pre-op and 6 weeks post-op. Data were analyzed using a paired *t* test, with the scatter dot plot representing both the mean values and individual data points (*n* = 23–39). DASH, Disability of Arm, Shoulder and Hand; pre-op; preoperation; post-op, postoperation.

Preoperative assessments indicated no significant differences in hand functionality between access and nonaccess limbs across all measured parameters (*P* > 0.05) (Figure 2). However, notable functional deficits emerged following hemodialysis access placement. Six weeks postsurgery, grip strength in the access limb decreased significantly compared with the nonaccess limb (*P* = 0.0002), with an average decrease of 5.8 pounds, representing an approximately 14% decline from preoperative grip strength (Figure 2a). Approximately 60% of patients experienced a decrease in grip strength^3^ 10% at 6 weeks following hemodialysis access surgery. Similar grip strength declines across access configurations indicated that early postoperative dysfunction was not explained entirely by access flow characteristics. Postoperatively, systolic finger pressure in the access arm significantly decreased by 30.6 ± 38.0 mm Hg (∼18% reduction) relative to preoperative levels (*P* < 0.0001) (Figure 2b). Hemodynamic changes were present in the wrist, where pressures of the radial and ulnar arteries were significantly lower postoperatively than the preoperative values (*P* < 0.05) (Supplementary Figure S3). Despite the presence of measurable hemodynamic alterations, none of the patients in the cohort experience hand ischemia that required surgical revision or therapeutic intervention. Patient-reported outcomes assessed using the DASH questionnaire indicated new or worsening hand and arm disabilities following vascular access surgery (*P* = 0.0213) (Figure 2c). In contrast, assessments of dexterity and digital sensation showed no significant postoperative changes (*P* > 0.05) (Figures 2d and e).

### Muscle Morphology Reveals Myofiber Atrophy Following Hemodialysis Access Placement

**Figure 3:**
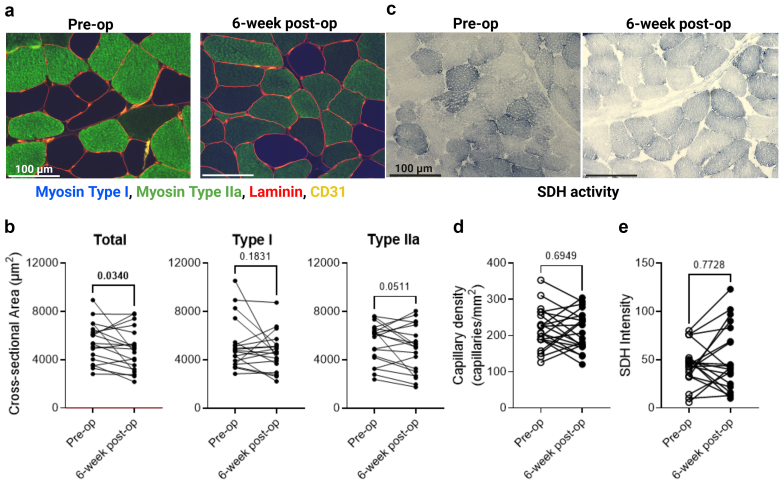
Myofiber cross-sectional area is lower 6 weeks following hemodialysis access placement. (a) Representative immunofluorescence images of brachioradialis muscle biopsies, showing labeled fiber types, membrane structures, and capillaries. (b) Cross-sectional areas of total, type i, and type IIa myofibers at pre-op and 6 weeks post-op (*n* = 19). (c) Representative images of SDH activity. (d) Quantification of capillary density at pre-op and 6 weeks post-op (*n* = 21). (e) Quantification of SDH activity in muscle sections at pre-op and 6 weeks post-op (*n* = 20). Data were analyzed using a ratio paired *t* test, with the scatter dot plot representing both the mean values and individual data points. pre-op; preoperation; post-op, postoperation; SDH, succinate dehydrogenase.

Using muscle biopsy specimens obtained from the brachioradialis muscle before and 6 weeks postoperatively, immunolabeling of the myofiber membrane with laminin revealed a significant reduction in the mean myofiber cross-sectional area, which was approximately 12% smaller at 6 weeks postoperatively compared with the preoperative condition (*P* = 0.0340) (Figure 3a and b). Analysis of the myofiber cross-sectional area by fiber type revealed that this myofiber atrophy was more pronounced in type IIa myofibers than type I myofibers, which showed no change (type I: *P* = 0.1831; type IIa: *P* = 0.0511). No differences were detected in capillary density (Figure 3a and d) or succinate dehydrogenase activity (Figure 3c and e) between the pre- and postoperative assessments.

### Relationship Between ARHD, Morphology, and Mitochondrial Function

**Figure 4:**
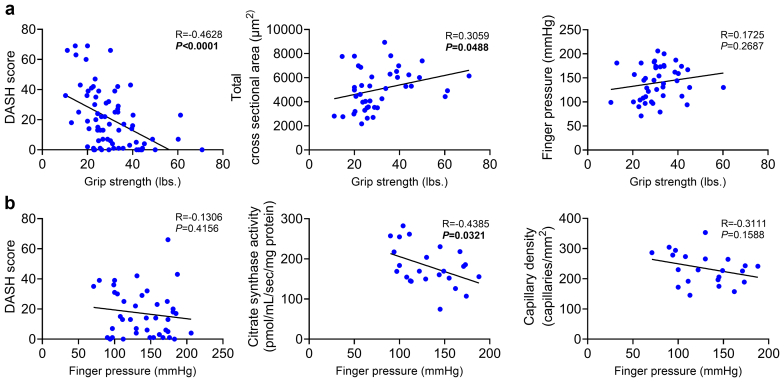
Relationship between primary functional outcomes. (a) Pearson correlations between grip strength and variables, including DASH score, mean cross-sectional area of total and type IIa myofibers, and finger pressure; as well as postoperative delta values between grip strength and finger pressure (*n* = 22–77). (b) Pearson correlations between finger pressure and variables, including DASH score, citrate synthase activity, maximum *J*O_2_, and capillary density; as well as postoperative delta values between finger pressure and DASH score (*n* = 21–49). Statistical analyses performed using 2-tailed Pearson correlation coefficient. DASH, Disability of Arm, Shoulder and Hand.

Grip strength showed a significant inverse correlation with DASH scores (*P* < 0.0001), indicating that lower grip strength was associated with worse perceived hand and arm disability (Figure 4a and Supplementary Figure S4A). In addition, grip strength positively correlated with both total (*P* = 0.0488) and type IIa myofiber cross-sectional area (*P* = 0.0211), indicating that myofiber atrophy is likely a significant contributor to the postoperative decrease in grip strength. Notably, finger pressure was not correlated with grip strength (*P* = 0.2687), or the change in grip strength (*P* = 0.3941). Similarly, finger pressure did not correlate with overall DASH scores (*P* = 0.4156) or the changes in DASH scores (*P* = 0.6531). These findings align with a previous report^4^ indicating that postoperative hemodynamic changes poorly correlate with hand dysfunction. Interestingly, finger pressure exhibited an inverse correlation with citrate synthase activity (*P* = 0.0321) and a trend toward an inverse correlation with maximal oxygen consumption rate (*P* = 0.0964) (Figure 4b and Supplementary Figure S4B), suggesting that even subtle postoperative hemodynamic changes in the access limb likely stimulate an adaptive mitochondrial biogenesis response. No significant correlation was found between finger pressure and capillary density (*P* = 0.1588), further highlighting the complex nature of functional and structural changes following vascular access surgery.

### Association Between ARHD and Unassisted Maturation of the Vascular Access

**Figure 5:**
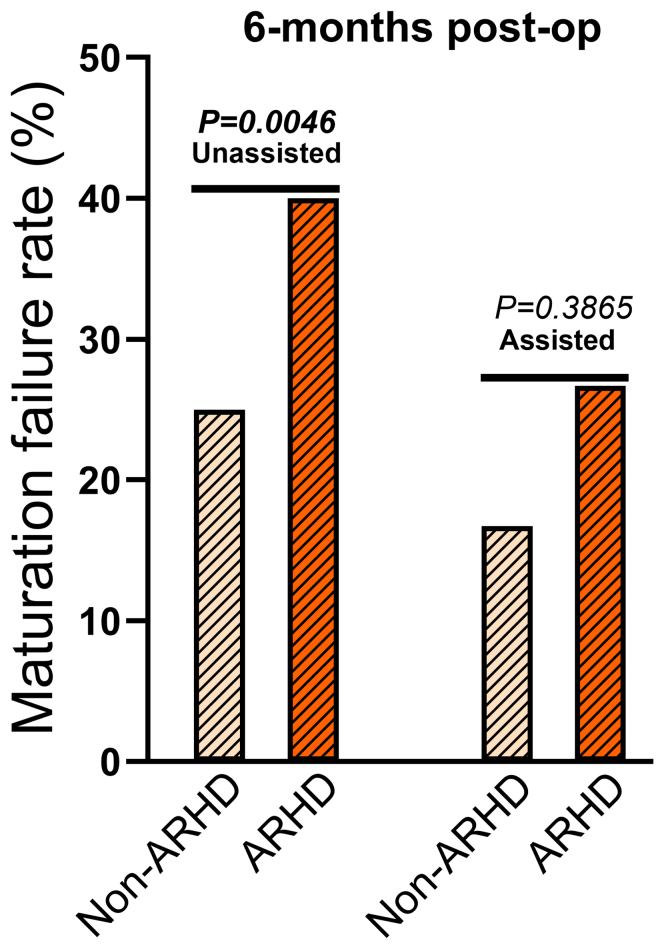
Patients with ARHD had greater access failure rates. Patients with ARHD, defined as a reduction in grip strength of > 10% from the preoperative value, exhibited significantly higher rates of unassisted maturation failure and a nonsignificant trend toward increased assisted maturation failure compared with patients without ARHD (*n* = 32). Statistical analysis performed using Chi-square test. ARHD, access-related hand dysfunction; post-op, postoperation.

At 6 months postoperatively, patients who experienced a >10% reduction in grip strength at 6 weeks had a significantly higher incidence of unassisted maturation failure than those with <10% change from baseline (*P* = 0.0046) (Figure 5). However, the incidence of assisted maturation failure was not significantly different between those with and without ARHD (*P* = 0.3865). Further analysis by access type revealed that patients with RC-AVF had higher rates of access failure than those with BC-AVF in both unassisted maturation failure (RC-AVF: 46.2% vs. BC-AVF: 25.0%, *P* = 0.0027) and assisted maturation failure (RC-AVF: 15.8% vs. BC-AVF: 6.3%, *P* = 0.0541). Importantly, the distribution of access types was similar between patients with and without ARHD (ARHD: 36.8% of RC-AVF, 52.6% of BC-AVF, 10.5% of AVG vs. non-ARHD: 46.2% of RC-AVF, 46.2% of BC-AVF, 7.7% of AVG, *P* = 0.381), suggesting that ARHD is not associated with access type or configuration. All access outcomes were assessed as categorical events occurring within the 6-month postoperative period rather that as time-to-event analyses.

### Changes in Skeletal Muscle Mitochondrial Function Following Hemodialysis Access Placement

Six weeks after hemodialysis access creation, state 2 respiration with pyruvate and malate showed a trend toward increased activity to preoperative levels (*P* = 0.0809); however, adenosine diphosphate–stimulated state 3 respiration with either pyruvate, octanoylcarnitine, or succinate showed no significant difference (Supplementary Figure S1A). A mitochondrial “stress test” assessing oxidative phosphorylation across varying energy demands revealed no differences in oxidative phosphorylation conductance between pre- and postoperative measurements (Supplementary Figure S1B) though citrate synthase activity (a mitochondrial content marker) tended to increase postoperatively (Supplementary Figure S1B). This suggests that the observed increase in state 2 mitochondrial respiration following hemodialysis access placement likely reflects increased mitochondrial content rather than alterations in bioenergetic function. Assessment of mitochondrial H_2_O_2_ production under substrate conditions identical to those used in the “stress test” revealed no significant differences in mitochondrial H_2_O_2_ production between pre- and postoperative conditions, regardless of energy demand (Supplementary Figure S1C). Furthermore, the estimated mitochondrial electron leak—calculated as the ratio of *J*H_2_O_2_ to *J*O_2_—showed no significant differences between pre- and postoperative conditions (Supplementary Figure S1C). Finally, we examined succinate-induced H_2_O_2_ emission and auranofin-induced H_2_O_2_ production to quantify reactive species (ROS) scavenged by the thioredoxin-peroxiredoxin antioxidant system. This analysis revealed no significant differences in either the capacity for H_2_O_2_ emission or production, as well as the percentage of scavenged H_2_O_2_. These findings suggest a stable mitochondrial H_2_O_2_ production and electron leak across different physiological states, indicating that hemodialysis access placement does not alter mitochondrial ROS homeostasis at 6 weeks postoperative time point examined in this study.

### Changes in Muscle Transcriptome Profiles Following Hemodialysis Access Placement

We performed unbiased RNA sequencing analysis on forearm muscle biopsies obtained preoperatively and 6 weeks postoperatively to assess how hemodialysis access placement alters the muscle transcriptome. Gene expression was quantified for 29,624 genes. Principal component analysis revealed significant overlaps between the pre- and postoperative transcriptomes (Supplementary Figure S2A). Compared with preoperative muscle, 69 differentially expressed genes (38 upregulated and 31 downregulated) were detected following hemodialysis access placement (Supplementary Figure S2A). Gene set enrichment analysis revealed that significantly upregulated genes were enriched in pathways related to “myoblast proliferation and differentiation” and “skeletal muscle fiber development”; whereas downregulated genes were enriched in pathways related to “response to glucocorticoid” and “regulation of muscle adaptation” (Supplementary Figure S2B). Full results of the Differentially Expressed Genes and Gene Set Enrichment Analysis analysis for each comparison can be found in Supplementary Dataset S1. Notably, several key genes involved in myogenesis, inflammation and fibrosis, and extracellular matrix remodeling exhibited upregulation following the surgical intervention (Supplementary Figure S2C, upper panels). In contrast, genes involved in metabolic functions and muscle atrophy demonstrated a marked downregulation posthemodialysis access creation (Supplementary Figure S2C, lower panels) despite significant myofiber atrophy being present (Figure 3).

### Subgroup Analysis of ARHD Based on Hemodialysis Status, Hemodialysis Access Site, and Race

We conducted subgroup analyses to evaluate key functional outcomes as it relates to preoperative hemodialysis status, vascular access location, and primary race. At the time of surgery, 56.4% of patients were receiving chronic hemodialysis using a tunneled dialysis catheter. A significant postoperative decrease in grip strength was observed in both nondialysis (*P* = 0.0251) and hemodialysis-dependent (*P* = 0.0054) patients (Supplementary Figure S5A). The relative postoperative change in grip strength was similar between nondialysis (−12.0%) and hemodialysis-dependent (−14.6%) patients. Patients not receiving hemodialysis reported greater changes in hand or arm disability postoperatively (Supplementary Figure S5B), despite hemodialysis-dependent patients having greater changes in finger pressure postoperatively (Supplementary Figure S5C). The greater change in finger pressure in patients receiving chronic hemodialysis treatment versus those not receiving hemodialysis treatment before enrollment is likely driven by differences in the proportion of access configurations in each group. The hemodialysis group consisted of 54.5% BC-AVFs, 27.3% RC-AVFs, and 18.2% AVGs whereas the nonhemodialysis group consisted of 40% BC-AVFs and 60% RC-AVFs with no patient in the nonhemodialysis group undergoing AVG placement.

It is possible that hemoaccess configuration could impact the severity of ARHD because of the differences in the rates of surgical remediation for steal syndrome between forearm and upper arm based dialysis access.^24^ To assess this possibility, we performed subgroup analysis according to the vascular access location: proximal upper arm (BC-AVF and AVG) versus distal forearm (RC-AVF). Both upper and lower arm groups experienced significant decreases in grip strength postoperatively (*P* = 0.0069 and *P* = 0.0207, respectively, Supplementary Figure S6A). In fact, the relative decrease in grip strength was comparable between upper arm (BC-AVF/AVG, −12.9%) and lower arm (RC-AVF, −14.4%) configurations. Similarly, both subgroups reported trends in increases in DASH scores (Supplementary Figure S6B) and exhibited a significant reduction in finger pressure (Supplementary Figure S6C).

Both Caucasian and African American patients experienced significant decreases in grip strength postoperatively (Supplementary Figure S7C), with the relative magnitudes being similar (−14.7% vs. −13.3% for Caucasian and African American, respectively). The changes in DASH scores were similar between racial groups (Supplementary Figure S7B); however, Caucasian patients experienced greater decreases in finger pressure postoperatively (Supplementary Figure S7C). Notably, the distribution of access configurations was similar between Caucasian (BC-AVF: 47.8%, RC-AVF: 47.8%, AVG: 4.3%) and African American patients (BC-AVF: 39.5%, RC-AVF: 46.2%, AVG: 23.1%).

## Discussion

The present study reveals that approximately 60% of patients with CKD or ESKD who undergo hemodialysis access placement develop ARHD postoperatively, characterized by significant reductions in grip strength and increases in perceived hand and arm disabilities. Histological analysis of the forearm muscle also demonstrates significant myofiber atrophy postoperatively. Exploratory transcriptomic profiling of skeletal muscle biopsies revealed marked alteration of genes involved in myofiber development, inflammatory processes, and mitochondrial metabolism, providing molecular context for the observed functional and structural impairments. Reaffirming previous observations that hemodynamic alterations alone do not completely explain ARHD,^4^ no significant correlation was found between digital hypoperfusion and other hand dysfunction indexes. To our knowledge, this is the first investigation to utilize skeletal muscle biopsies to characterize morphology and molecular features underlying ARHD.

Grip strength, a validated predictor of morbidity, served as the primary indicator for clinical interpretation. A notable finding in the present study was a significant decline in grip strength, which is closely associated with forearm muscle fiber atrophy following hemodialysis access placement. Approximately 70% of patients experienced a reduction in grip strength, with an average decline of 14%, whereas muscle cross-sectional area decreased by 12% compared with preoperative baselines. This finding highlights the role of muscle atrophy as a key contributor to the functional impairments observed in the access hand or arm. Accelerated muscle wasting and loss of strength are common manifestations in CKD,^25^, ^26^, ^27^ and > 50% of patients on hemodialysis report preoperative hand or arm disabilities compared with age-matched, nondialysis counterparts.^7^^,^^28^ Notably, these functional deficits are exacerbated by hemodialysis access placement determined here by patient reporting of new or worsening hand and upper extremity disabilities postoperatively. Given that reduced muscle strength is closely linked to increased morbidity and mortality,^29^^,^^30^ further compromising patients’ quality of life^10^, ^11^, ^12^ and that dialysis patients face a mortality rate approximately 10 times higher than age-matched Medicare patients without kidney disease,^31^ losses in muscle mass and strength following hemodialysis access formation should not be overlooked. Although the absolute decline in grip strength (∼5 lbs) may appear modest, even small declines have been associated with reduced daily functional capacity and higher mortality risk in CKD and community cohorts.^4^^,^^10^, ^11^, ^12^^,^^21^^,^^29^^,^^30^^,^^32^ However, minimal clinically important difference for grip strength has not been established in CKD or hemodialysis populations.^33^, ^34^, ^35^ Therefore, the clinical significance of the magnitude of change (∼14% reduction) reported in this study should be interpreted with caution.

A significant correlation between changes in perceived hand and arm disability and grip strength support the notion that muscle weakness directly contributes to diminished ability to perform routine activities of daily living and remain functional independent, leading to a poor quality of life.^36^, ^37^, ^38^ Along with this, RNA sequencing data determined upregulation of myogenic genes and downregulation of atrophy-related genes, potentially indicative of compensatory molecular adaptations aimed at counteracting muscle atrophy and weakness in the access arm.^39^^,^^40^ These findings suggest that proactive interventions to mitigate muscle atrophy and strength loss in the perioperative period of vascular access surgery may be essential for preserving postoperative functional capacity of the access hand or arm. Interestingly, despite the obvious functional declines following hemodialysis access formation, measures of digital sensation and dexterity did not show significant postoperative differences, indicating that the fine motor control and sensory function remain relatively preserved, at least in the early stages postsurgery or the methods for assessment may not be sensitive enough to reveal the subtle changes. This aligns with a previous report^4^ indicating that whereas gross motor functions such as grip strength are more directly affected, sensory and dexterous functions tend to decline more gradually or may reflect the different impairment thresholds among motor and sensory nerve fibers and muscle cells, or the impact of preexistent polyneuropathy or myopathy.^7^^,^^41^, ^42^, ^43^

Hemodynamic perturbations associated with clinically overt steal syndrome is a well-established complication associated with vascular access placement.^18^^,^^23^^,^^44^ Consistent with previous studies, we observed a mean reduction of approximately 31 mm Hg in finger pressures following vascular access placement. However, none of the individuals in this cohort exhibited ischemic complications severe enough to warrant surgical or therapeutic management, suggesting that the hemodynamic changes commonly observed following vascular access creation are subclinical. Furthermore, there were no significant correlations between finger pressure and either grip strength or perceived hand or arm disabilities. This observation aligns with previous findings^4^ and challenges the prevailing assumption that hemodynamic changes are the predominant driver underlying ARHD. Subclinical digital hypoperfusion accounts for only a fraction of the measured postoperative hand dysfunction, supporting that other multifactorial muscular and neurovascular mechanisms play an important role in the etiology of ARHD.

We observed a significant inverse correlation between finger pressure and citrate synthase activity, indicating that adaptive responses to the access-related hemodynamic stress stimulates mitochondrial biogenesis in the access arm.^45^^,^^46^ However, this finding does not reflect improved mitochondrial efficiency. Adenosine diphosphate–stimulated maximal mitochondrial respiration per unit of muscle wet weight under multisubstrate conditions was not different between pre- and postoperative states. Furthermore, no significant differences were observed in mitochondrial function, including oxidative phosphorylation capacity, electron leak, or antioxidant capacity. The absence of a net improvement in mitochondrial function may suggest a maladaptive response to ischemic stress in these patients. The observed increased tendency in mitochondrial content without changes in mitochondrial respiration suggests an adaptive but inadequate response to postoperative hemodynamic stress. This notion is evidenced by the downregulated genes associated with metabolic adaptation to energetic demand, along with unchanged succinate dehydrogenase intensity.

Subgroup analyses of key functional outcomes determined a significant decline in grip strength regardless of chronic hemodialysis, access location, or race. However, perceived limb disability was greater among patients not receiving hemodialysis, whereas the reduction in finger pressure was more pronounced in patients on hemodialysis compared with their baseline levels, despite no statistical difference in each hand dysfunctional outcomes between groups. This difference could be related to patients on current chronic hemodialysis receiving a greater proportion of upper arm hemoaccess configurations. However, subgroup analysis based on upper versus lower arm indicated that both groups suffer from similarly modest decreases in finger pressure postoperatively. A postoperative decrease in grip strength was comparable between Caucasian and African American patients, suggesting that a racial disparity may not be present for ARHD. However, it is worth noting that these subgroup analyses are likely underpowered and larger confirmatory studies are needed.

In the current cohort, we identified a difference in the proportion of patients who experience unassisted maturation failure between patients that suffered from ARHD (a > 10% decrease in grip strength) versus those patients whose grip strength changes were < 10%. Notably, a higher incidence of unassisted maturation failure among patients with ARHD raises the possibility that more pronounced functional impairments negatively influence vascular access outcomes. Although these observations do not establish a causal relationship, this finding aligns with previous studies linking upper extremity dysfunction to altered hemodynamics and increased mechanical stress around the access site, which may affect access adaptation and patency over time.^47^ Given the multifactorial etiology of postoperative AHRD, perioperative implementation of interventions aimed at concurrently enhancing muscle and vascular health. Future studies should focus on elucidating the mechanisms by which postoperative functional decline contributes to access failure and evaluate whether targeted interventions can improve long-term access maturation and patient quality of life. In this regard, structured perioperative hand-exercise regimens, which have been previously reported with variable results to augment AVF maturation,^48^, ^49^, ^50^ may mitigate muscle atrophy and risk of development of ARHD. However, the heterogeneity in protocols, timing, and measured outcomes highlights the need for more rigorous prospective trials to determine true clinical benefit of perioperative exercise in this population.

### Limitations

This study provides valuable insights into the molecular etiology of AHRD and its relationship with arteriovenous access outcomes; however, several limitations should be considered. First, the study involves a modest sample size, which may limit statistical power and the detection of subtle associations or subgroup differences; thus highlighting the need for a larger cohort to enhance the robustness of our findings. Our cohort was predominantly male and included a high proportion of African American participants; therefore, larger more diverse studies are warranted, although the uniformity of functional decline across groups suggests conserved mechanisms. Although frailty was not formally assessed, adjusting for preexisting surrogate markers, including body weight and body mass index, did not affect the observed differences in grip strength—the primary ARHD outcome—indicating robustness of the current findings. Nonetheless, the uniformity of functional decline across subgroups suggests conserved mechanisms. Second, the use of muscle biopsies exclusively from the access arm limits our ability to evaluate contralateral differences, which could clarify whether the observed muscle atrophy and transcriptomic profiles are localized to the access arm or indicative of a systemic response to surgery or metabolic alterations. In addition, muscle specimens were only obtained from the brachioradialis muscle and it remains unknown of other muscles in the forearm or hand respond similarly to hemodialysis access placement. However, it is worth noting that obtaining percutaneous biopsies from multiple hand/arm muscles is a challenging endeavor. Third, the absence of longitudinal flow measurements limits our ability to directly evaluate early access (physiological) maturation failure rates of RC-AVF and BC-AVF by access flow. Incorporating longitudinal flow assessments in future studies may provide a more comprehensive understanding of hemodynamic changes over time and their potential impact on access patency and hand dysfunction. Nonetheless, it is important to note that finger and wrist blood pressure changes did not correlate with the changes in grip strength or perceived hand and arm disability. Fourth, this study focused on changes of ARHD parameters only in 6 weeks postoperation and does not fully capture either the early adaptive responses or the longer-term effects on ARHD. Although previous studies report minimal change in grip strength and only partial recovery of perfusion between 3 and 6 months postoperatively,^4^^,^^21^ our functional assessments were limited to the 6-week time point. Therefore, potential improvements associated with subsequent vascular remodeling cannot be excluded. Implementing a more comprehensive time-course analysis could facilitate the identification of critical early and late adaptations. However, this approach would increase the burden on the patients, which can diminish enrollment and completion of the study protocol, especially if including forearm muscle biopsy procedures.

## Conclusion

This study demonstrates that up to approximately 60% of patients with CKD or ESKD develop ARHD following hemodialysis access placement, characterized by muscle weakness, distal hypoperfusion, and perceived hand and arm disabilities. Morphological analysis and correlation data suggest that the decline in grip strength is mainly driven by muscle atrophy. Supporting this, postoperative transcriptomic profiles reveal that most differentially expressed genes were associated with myofiber development pathways. Mitochondrial assays suggest that distal hypoperfusion is associated with increased mitochondrial content, but not with a corresponding improvement in mitochondrial functional capacity. Moreover, the absence of a significant correlation between grip strength or perceived hand or arm disability and finger pressure indicates that ARHD is driven by multifactorial condition. The association between ARHD and high incidence of vascular access maturation failure highlights the potential of hand or arm dysfunction as an early clinical indicator of vascular access outcomes. Collectively, these findings provide new insights into the potential mechanisms underlying ARHD and suggest that both vascular and muscular factors contribute to postoperative hand dysfunction. Although our study helps narrow the existing knowledge gap, further research is warranted to fully elucidate the pathophysiology of ARHD and to develop effective, targeted interventions that improve access outcomes and patient quality of life.

## Disclosure

All the authors declared no competing interests.

## Supplementary Material

Supplementary File (PDF) Supplementary Methods. Detailed experimental methods. Supplementary References. Figure S1. Mitochondrial function. Figure S2. Transcriptomic profiles. Figure S3. Radial and ulnar artery pressure. Figure S4. Pearson correlation. Figure S5. Subgroup analysis of ARHD based on hemodialysis status. Figure S6. Subgroup analysis of ARHD based on access site. Figure S7. Subgroup analysis of ARHD based on race. Screening questionnaire. DASH questionnaire.

## Footnotes

Supplementary File (PDF)
**Supplementary Methods.** Detailed experimental methods.
**Supplementary References.**
**Figure S1.** Mitochondrial function. **Figure S2.** Transcriptomic profiles. **Figure S3.** Radial and ulnar artery pressure. **Figure S4.** Pearson correlation. **Figure S5.** Subgroup analysis of ARHD based on hemodialysis status. **Figure S6.** Subgroup analysis of ARHD based on access site. **Figure S7.** Subgroup analysis of ARHD based on race. Screening questionnaire. DASH questionnaire.
