# Supplementary material for: Access-Related Hand Dysfunction After Hemodialysis Access Placement
Source: Kidney Int Rep. 2026 Jan 2;11(3):103765. doi: 10.1016/j.ekir.2025.103765 (PMC12906999; doi:10.1016/j.ekir.2025.103765)
Supplement: Supplementary File (PDF) — Supplementary Methods. Detailed experimental methods. Supplementary References. Figure S1. Mitochondrial function. Figure S2. Transcriptomic profiles. Figure S3. Radial and ulnar artery pressure. Figure S4. Pearson correlation. Figure S5. Subgroup analysis of ARHD based on hemodialysis status. Figure S6. Subgroup analysis of ARHD based on access site. Figure S7. Subgroup analysis of ARHD based on race. Screening questionnaire. DASH questionnaire. [file mmc1.pdf]

Access-related Hand Dysfunction after Hemodialysis Access Placement

Kyoungrae Kim[1], Trace Thome[1], Lauren Stone[1], Nicholas Vugman[1], Eric M. Kunz[1], Samuel Alvarez[1], Qingping Yang[1], Kerri A. O'Malley[2], Erik M. Anderson[2], Brian Fazzone[2], Pavel Mazirka[2], Jesseca Antoine[2], Scott A. Berceli[2,3], Terence E. Ryan[1,4,5], Salvatore T. Scali[2, 3]

[1]Department of Applied Physiology and Kinesiology
[2]Division of Vascular Surgery and Endovascular Therapy
[3]Malcom Randall Veterans Affairs Medical Center
[4]Center for Exercise Science
[5]Myology Institute
University of Florida, Gainesville, FL, USA

[#]**Correspondence:** Salvatore T. Scali, MD: 1600 SW Archer Rd, Gainesville, FL, 32608. Tel: 352-376-1611 (office); email: salvatore.scali@surgery.ufl.edu

Supplemental Methods

Sex as a biological variable

This study enrolled both male and female participants; however, sex was not considered as a biological variable in the study design.

Inclusion Criteria

The inclusion criteria specified that adult patients have a minimum life expectancy of nine months and were either undergoing hemodialysis or expected to begin chronic hemodialysis within three months of surgery. Additionally, the inclusion criteria were restricted to patients undergoing first-time hemodialysis access in the ipsilateral arm, excluding those with prior AV access attempts to minimize residual effects of AV-access associated hand dysfunction from prior surgeries.

Operative procedures and postoperative follow-up

All patients underwent preoperative, noninvasive arterial and venous mapping of the upper extremity via duplex ultrasound to determine the most suitable vascular configuration for creating hemodialysis access, in accordance with established guidelines[S1-4]. The vascular access was placed on the side with the most suitable vein (e.g., ≥3mm diameter), irrespective of the patient's hand dominance. Hemodialysis access configurations included the radio-cephalic AVF in the distal forearm, brachial-cephalic AVF in the upper arm, and brachio-axillary bridge/ axillary-to-axillary upper arm loop graft. These procedures were performed under local or general anesthesia with conscious sedation, with no upper extremity regional anesthetic blocks being administered during the surgeries. Postoperative monitoring was conducted to evaluate access failure, with detailed data collected on fistula patency and potential complications such as stenosis, thrombosis, and infection.

Grip strength test

A modified Groningen Elderly Test protocol[S5] was utilized to evaluate grip strength in patients undergoing hemodialysis access placement using a Smedley Digital Hand Dynamometer (Model 12-0286, Baseline Evaluation Instruments, China). In a standing position, patients were instructed to rest their hand at their side, hold the dynamometer, and squeeze it as hard as possible. Patients were provided with one practice trial to familiarize with the procedure, followed by three attempts with 30-second rest intervals between each attempt to minimize fatigue and ensure consistent performance across trials. Both extremities were tested in a randomized order, and the highest value in pounds (lbs.) achieved across the three trials was recorded.

Finger and wrist pressure test

Systolic pressure was measured bilaterally on the index finger, radial artery, and ulnar artery at the wrist to evaluate the extent of peripheral perfusion as previously described[S1]. The measurements were taken using inflatable cuffs and 4- to 8-MHz Doppler probes (Parks Flow Lab, Aloha, OR), placed at an angle of 45° to 60° relative to the expected longitudinal axis of the arteries. The cuffs were inflated to 20-30 mmHg above the last audible Doppler signal and deflated automatically, with the systolic pressure recorded at the first reappearance of the Doppler sound. To avoid potential interference with hemodynamic assessments, patients were instructed to abstain from taking antihypertensive medication 24 hours before both pre- and post-operative visits. For patients with radiocephalic fistulas, wrist pressure measurements were omitted, and only digital pressures were recorded.

Perceived access hand/arm disabilities

Patient perception of access hand/arm function and symptomatology with routine daily tasks was evaluated using the Disabilities of the Arm, Shoulder and Hand (DASH) questionnaire, which is a validated survey to assess general limb disability[S6, 7]. The questionnaire consists of 30 questions answered on a 5-point Likert scale where 1 indicates no difficulty (or no pain) and 5 indicates unable to perform (or extreme pain). The recorded scores for all items were summed to obtain a total raw score. The DASH score was then calculated using the following formula: DASH score= [(sum of n responses/n)-1] x 25, where n is equal to the number of responses.

Dexterity

The Purdue Pegboard test was used as a validated tool to assess motor coordination and hand and digit dexterity[S8]. A patient's score equaled the average among 3 consecutive trials of the number of pegs properly placed in the pegboard (Model 3200, Lafayette Instrument, Lafayette, IN) within a 30-second period.

Sensation

Light touch sensation at the first and fifth digits was evaluated using the standardized Semmes-Weinstein Monofilament testing method, employing tactile monofilaments with weights of 300g, 4.0g, 2.0g, 0.4g, and 0.07g (Tactile Monofilaments, Baseline Evaluation Instruments, Fabrication Enterprises Inc., White Plains, New York)[S9]. During the assessment, the patient's eyes were closed, and the research coordinator applied the monofilament to the ventral surface of the fingertip three times at intervals of 1.5 seconds. The order of applications was randomized to minimize bias, and the lowest-weight monofilament that the patient could detect was recorded. The resulting raw data were then transformed so that a score of 1 corresponded to the 300g monofilament (worst sensation) and 5 corresponded to the 0.07g monofilament (best sensation).

Forearm muscle biopsy

Pre- and post-operative muscle biopsies (~50 mg) were obtained from the brachioradialis muscle, positioned 5 to 10 cm distal to the cubital fossa, utilizing a 14-gauge biopsy needle. Following the biopsy, the specimens were immediately placed in ice-cold Buffer X (containing 7.23 mM K2EGTA, 2.77 mM CaK2EGTA, 20 mM Imidazole, 20 mM Taurine, 5.7 mM Na-ATP, 14.3 mM Na-PCr, 6.6 mM MgCl2-6H2O, and 50 mM MES potassium salt, pH 7.1) to ensure optimal preservation of the tissue. To ensure the accuracy and reliability of the analytical results, residual blood, connective tissue, and adipose tissue were carefully removed under the microscope. The cleaned muscle biopsy samples were then subjected to three distinct preparation techniques: they were snap-frozen for subsequent molecular analysis, embedded in optimal cutting temperature (OCT) compound and frozen in liquid nitrogen cooled isopentane for morphological evaluation, and prepared as myofiber bundles for mitochondrial functional assays.

Assessment of mitochondrial function

Muscle fibers designated for mitochondrial analysis were mechanically separated into bundles using fine-tipped forceps under a microscope. These bundles were permeabilized with 30 µg/mL saponin for 30 minutes at 4°C on an orbital shaker to allow for free substrate diffusion. After permeabilization, the fibers were washed in ice-cold buffer D (105 mM K-MES, 30 mM KCl, 1 mM EGTA, 10 mM K2HPO4, 5 mM MgCl2·6H2O, 0.5 mg/mL bovine serum albumin, pH 7.1) for 15 minutes to remove residual saponin. Thereafter, the wet weight of each myofiber bundle was recorded after blotting the sample (~10 seconds) to normalize oxygen consumption rates.

Maximum respiratory capacity was measured using the high-resolution Oroboros Oxygraph-2K respirometry system set at 37°C, in a mitochondrial assay buffer D supplemented with 20 mM creatine monohydrate. Prior to initiating the respiratory assay, 0.01 mM blebbistatin, a myosin inhibitor, was added to prevent involuntary muscle contractions[S10]. After permeabilized myofiber bundles (~3 mg) were placed in the respirometry chamber, the assay buffer was hyper-oxygenated to ~300 µM to preclude oxygen diffusion limitations. Once a stable baseline in oxygen flux was achieved, mitochondria were energized by adding 2.5 mM malate and 5 mM pyruvate (State 2). Subsequently, 4 mM ADP was introduced to stimulate maximum respiratory capacity (State 3). To promote complex I-associated respiration, 0.2 mM octanoylcarnitine was added, followed by 10 mM succinate to measure complex II-associated respiration. Finally, 0.005 mM cytochrome c was added to assess mitochondrial membrane integrity, with data showing greater than 10% increase in maximum respiration being excluded from the final analysis to ensure the integrity of the muscle bundle separation process.

Oxygen flux (JO_2) in buffer D supplemented with 5mM creatine monohydrate was assessed to simulate physiological conditions using a creatine kinase clamp system[S11], which allows for measurement of JO_2 across a range of ATP-free energy states from

near-resting conditions to maximum contractions. Initially, permeabilized myofibers were energized with 5 mM pyruvate, 2.5 mM malate, and 0.2 mM octanoylcarnitine, and then the creatine kinase clamp was introduced by adding 20 U/ml creatine kinase, 5 mM ATP, and 1 mM phosphocreatine. Additional phosphocreatine was subsequently added in steps to reduce energy demand, replicating submaximal contractions and resting condition. The slope of the relationship between energy demand (ΔG_{ATP}) and JO_2, referred to as OXPHOS conductance, was determined.

Mitochondrial hydrogen peroxide (H_2O_2) emission corresponding to the OXPHOS during the metabolic stress test or saturated succinate-driven H_2O_2 production was assessed using a Amplex UltraRed/horseradish peroxidase detection system (Horiba Fluorolog) as described previously[S12]. Fluorescence spectroscopy was carried out using a Quantmaster-400 (Horiba Instruments) with excitation at 565 nm and emission at 590 nm. The fluorescence data were converted into picomoles of H_2O_2 using a standard curve. To estimate electron leak, H_2O_2 flux ($J\ H_2O_2$) was divided by JO_2 under each identical substrate condition.

Citrate synthase activity

Citrate synthase (CS) activity was assessed using a commercially available assay kit (Sigma-Aldrich, CS0720). Approximately 5mg snap frozen muscle specimens were manually homogenized in Celytic M Cell Lysis Reagent (Sigma-Aldrich, C2978) including protease and phosphatase inhibitors (Millipore Sigma, P8340 and 524627, respectively with a ratio of 1:100). This was accomplished using 1 ml glass pestle tissue grinders under ice-cold conditions (Corning Pyrex, 7724-1). Following the centrifugation at 12,000g for 15 min at 4°C, aqueous supernatants were transferred into clean tubes, and protein concentrations were determined using a Pierce Rapid Gold BCA protein assay kit (Thermo Fisher Scientific, A53225). After normalizing the protein amount and volume with the lysis buffer (1 µg/µl), 10 µg of protein per well was loaded, and CS activity measurements were conducted in an assay buffer consisting of 100 mM Tris, 1 mM EDTA, 10 mM DTNB, and 30 mM acetyl CoA at pH 8.35. Absorbance readings were taken at 412 nm at 37°C. Endogenous absorbance was measured for 4 minutes before the addition of 10 mM oxaloacetate, and total absorbance was recorded for an additional 4 minutes following the oxaloacetate addition. The CS activity was calculated as net absorbance by subtracting endogenous activity from total activity using the equation: CS activity (pmol/ml/sec/mg) = $[\Delta A_{412}/\text{sec} \times V\ (\text{ml}) \times \text{dil}]\ /\ [\varepsilon^{mM} \times L\ (\text{cm}) \times V_{enz}\ (\text{ml})]\ /\ [\text{mg}]$, where ΔA_{412}/min is the net absorbance; V is the reaction volume for 96-well plate (0.2 ml); dil is the dilution factor of the original sample (1); ε^{mM} is the extinction coefficient of TNA at 412 nm (13.6 $mM^{-1}cm^{-1}$); L is the pathlength of the well in 96-well plate (0.552 cm); V_{enz} is the sample volume (0.01 ml); mg is the amount of protein lysate.

Muscle histopathology

The evaluation of skeletal muscle fiber cross-sectional area (CSA) using immunofluorescence microscopy techniques. Frozen transverse sections of muscle tissue (5 μm thick) were cut and mounted onto microscope slides, followed by a 90-minute incubation in an Animal-Free blocking solution (Vector Laboratories, Cat. No. SP-5035-100). To label the sarcolemma, the sections were incubated overnight at 4°C with a primary antibody against laminin (Developmental Studies Hybridoma Bank, University of Iowa, Cat. No. 2E8, diluted 1:100) and primary antibodies targeting myosin heavy chain (MyHC) isoforms: MyHC I (Cat. No. BA-D5, diluted 1:100), MyHC IIa (Cat. No. SC-71 diluted 1:500), and CD31/PECAM1 (Abcam, Cat. No. ab28364, diluted 1:50). Following this, the slides were washed four times (5 minutes each) with phosphate-buffered saline (PBS) and subsequently incubated for one hour at room temperature with appropriate Alexa-Fluor-conjugated secondary antibodies (Cat. Nos. A21140, A21121, A21137, A32733; all diluted 1:300). After another round of washes in 1× PBS, coverslips were applied using Vectashield hard mount (Vector Laboratories, Cat. No. H-1400). Imaging was conducted at ×20 magnification using an Evos FL2 Auto microscope (ThermoFisher Scientific), capturing tiled images of the entire muscle for further analysis. Entire cross-sections were analyzed, with a mean of 179 fibers per sample (ranging from 42-365 fibers); investigators were blinded to pre- and postoperative status. Myofiber type specific CSA was identified through myosin heavy chain and laminin staining, and the quantification of myofiber CSA were carried out using Myosight, an automated macro developed in Fiji/ImageJ. To assess capillary density, the tiled images were thresholded in ImageJ/Fiji and counted by a blinded investigator utilizing coded images, with vessel density normalized to the muscle section area. For succinate dehydrogenase (SDH) activity staining, unfixed sections were allowed to air dry for five minutes before being immersed in a coplin jar containing a reaction mixture composed of 130 mM sodium succinate, 0.2 mM phenazine methosulfate, 1 mM sodium azide, and 1.5 mM nitrotetrazolium blue chloride (in 0.2 M phosphate buffer, pH 7.0) for 60 minutes at 37°C. Following the reaction, the slides were washed twice with 1× PBS at room temperature, dehydrated through a series of increasing ethanol concentrations, and cleared with xylenes prior to mounting with coverslips. Transmitted light images were captured at ×20 magnification using the Evos FL2 Auto microscope (ThermoFisher Scientific), and the mean intensity was employed to quantify SDH abundance and activity.

Bulk RNA sequencing

Total RNA was isolated from the brachioradialis muscle using the Direct-zol RNA MiniPrep kit (Zymo Research, Cat. No. R2052), in accordance with the manufacturer's protocol. RNA integrity was verified using RIN values, and only samples with RIN >7.5 were included. Library preparation and mRNA sequencing, employing PolyA selection, were subsequently performed by Genewiz (Azenta Life Science, South Plainfield, NJ). Paired-end sequencing was conducted using 150 bp reads on the Illumina HiSeq 4000

platform. To ensure data quality, sequence reads were trimmed for adapter contamination and low-quality bases using Trimmomatic v.0.36. The clean reads were then aligned to the Homo sapiens GRCh38 reference genome (ENSEMBL) via the STAR aligner v.2.5.2b. Gene hit counts were quantified using feature Counts from the Subread package v.1.5.2, targeting only unique reads that mapped to exon regions. These gene counts were subsequently used for differential expression analysis. Genes with fewer than 10 counts were excluded to minimize noise and improve biological relevance. DESeq2 was employed to conduct pairwise comparisons of gene expression between pre- and post-operative samples, generating *P*-values and log2 fold changes using the Wald test. Differentially expressed genes were identified based on an adjusted *P*-value of < 0.05 and an absolute log2 fold change > 0.5. This threshold was selected based on prior studies showing that even modest changes in transcript levels can represent biologically meaningful regulatory effects in *in vivo* tissues[S13-15]. Furthermore, gene set enrichment analysis (GSEA) was performed using GSEAPY.

Relationship between ARHD and vascular access failure

For an ancillary purposes, we investigated the relationship between changes in grip strength at six weeks post-operation and the rate of hemodialysis access failure at six months post-operation. Patients exhibiting a decline in grip strength greater than 10% from preoperative baseline were classified as having ARHD, while those whose grip strength decreased by less than 10% or showed improvement were categorized as the non-ARHD group. Hemodialysis access was evaluated through a comprehensive assessment that included physical examinations, duplex ultrasound imaging, and dialysis adequacy metrics. This multifaceted approach enabled a direct comparison between functional outcomes, as reflected by grip strength, and the success of hemodialysis access, providing insight into potential associations between these two clinical outcomes.

Statistical analysis

Normality of the data was assessed using Shapiro–Wilk test and visual inspection of QQ plots. For hand and arm functional measurements, paired t-tests were utilized to compare outcomes between the access and non-access limbs, as well as between pre- and post-operative conditions. Muscle histopathological evaluations, which considered variability in fiber counts, were analyzed using ratio paired t-tests to ensure accurate quantification of changes. A mixed-effects model was applied for repeated measures data, and Šídák's post hoc test was conducted for multiple comparisons when significant interactions were detected. To assess relationships between two continuous variables, Pearson correlation analysis was employed. Multivariable linear models were employed to evaluate the impact of ethnicity, dialysis status, and hemodialysis access configuration as covariates on ARHD. For bulk RNA sequencing data, the Benjamini–Hochberg method was used to calculate false discovery rate corrected *P*-values. To compare the

proportions of vascular access failure between patients with and without ARHD, a Chi-square test was used via VassarStats. All statistical analyses were performed using R-Studio and GraphPad Prism (v.9.0), with a *P*-value of less than 0.05 considered statistically significant. Data are presented as means ± standard deviation (SD) unless otherwise stated.

Supplemental Reference

S1. Huber TS, Ozaki CK, Flynn TC, Lee WA, Berceli SA, Hirneise CM, Carlton LM, Carter JW, Ross EA and Seeger JM. Prospective validation of an algorithm to maximize native arteriovenous fistulae for chronic hemodialysis access. *J Vasc Surg*. 2002;36:452-9.

S2. Vascular Access Work G. Clinical practice guidelines for vascular access. *Am J Kidney Dis*. 2006;48 Suppl 1:S248-73.

S3. Vascular Access Work G. Clinical practice guidelines for vascular access. *Am J Kidney Dis*. 2006;48 Suppl 1:S176-247.

S4. Sidawy AN, Spergel LM, Besarab A, Allon M, Jennings WC, Padberg FT, Jr., Murad MH, Montori VM, O'Hare AM, Calligaro KD, Macsata RA, Lumsden AB, Ascher E and Society for Vascular S. The Society for Vascular Surgery: clinical practice guidelines for the surgical placement and maintenance of arteriovenous hemodialysis access. *J Vasc Surg*. 2008;48:2S-25S.

S5. Lemmink KA, Han K, de Greef MH, Rispens P and Stevens M. Reliability of the Groningen Fitness Test for the Elderly. *Journal of Aging & Physical Activity*. 2001;9.

S6. Hudak PL, Amadio PC and Bombardier C. Development of an upper extremity outcome measure: the DASH (disabilities of the arm, shoulder and hand) [corrected]. The Upper Extremity Collaborative Group (UECG). *Am J Ind Med*. 1996;29:602-8.

S7. Gummesson C, Atroshi I and Ekdahl C. The disabilities of the arm, shoulder and hand (DASH) outcome questionnaire: longitudinal construct validity and measuring self-rated health change after surgery. *BMC musculoskeletal disorders*. 2003;4:1-6.

S8. Tiffin J and Asher EJ. The Purdue pegboard; norms and studies of reliability and validity. *J Appl Psychol*. 1948;32:234-47.

S9. Gelberman RH, Szabo RM, Williamson RV and Dimick M. Sensibility testing in peripheral-nerve compression syndromes. An experimental study in humans. *JBJS*. 1983;65:632-638.

S10. Perry CG, Kane DA, Lin CT, Kozy R, Cathey BL, Lark DS, Kane CL, Brophy PM, Gavin TP, Anderson EJ and Neufer PD. Inhibiting myosin-ATPase reveals a dynamic range of mitochondrial respiratory control in skeletal muscle. *The Biochemical journal*. 2011;437:215-22.

S11. Fisher-Wellman KH, Davidson MT, Narowski TM, Lin CT, Koves TR and Muoio DM. Mitochondrial Diagnostics: A Multiplexed Assay Platform for Comprehensive Assessment of Mitochondrial Energy Fluxes. *Cell reports*. 2018;24:3593-3606 e10.

S12. Berru FN, Gray SE, Thome T, Kumar RA, Salyers ZR, Coleman M, Dennis L, O'Malley K, Ferreira LF, Berceli SA, Scali ST and Ryan TE. Chronic kidney disease exacerbates ischemic limb myopathy in mice via altered mitochondrial energetics. *Sci Rep*. 2019;9:15547.

S13. Love MI, Huber W and Anders S. Moderated estimation of fold change and dispersion for RNA-seq data with DESeq2. *Genome biology*. 2014;15:550.

S14. Chen G, Ning B and Shi T. Single-cell RNA-seq technologies and related computational data analysis. *Frontiers in genetics*. 2019;10:317.

S15. Schaum N, Lehallier B, Hahn O, Pálovics R, Hosseinzadeh S, Lee SE, Sit R, Lee DP, Losada PM and Zardeneta ME. Ageing hallmarks exhibit organ-specific temporal signatures. *Nature*. 2020;583:596-602.

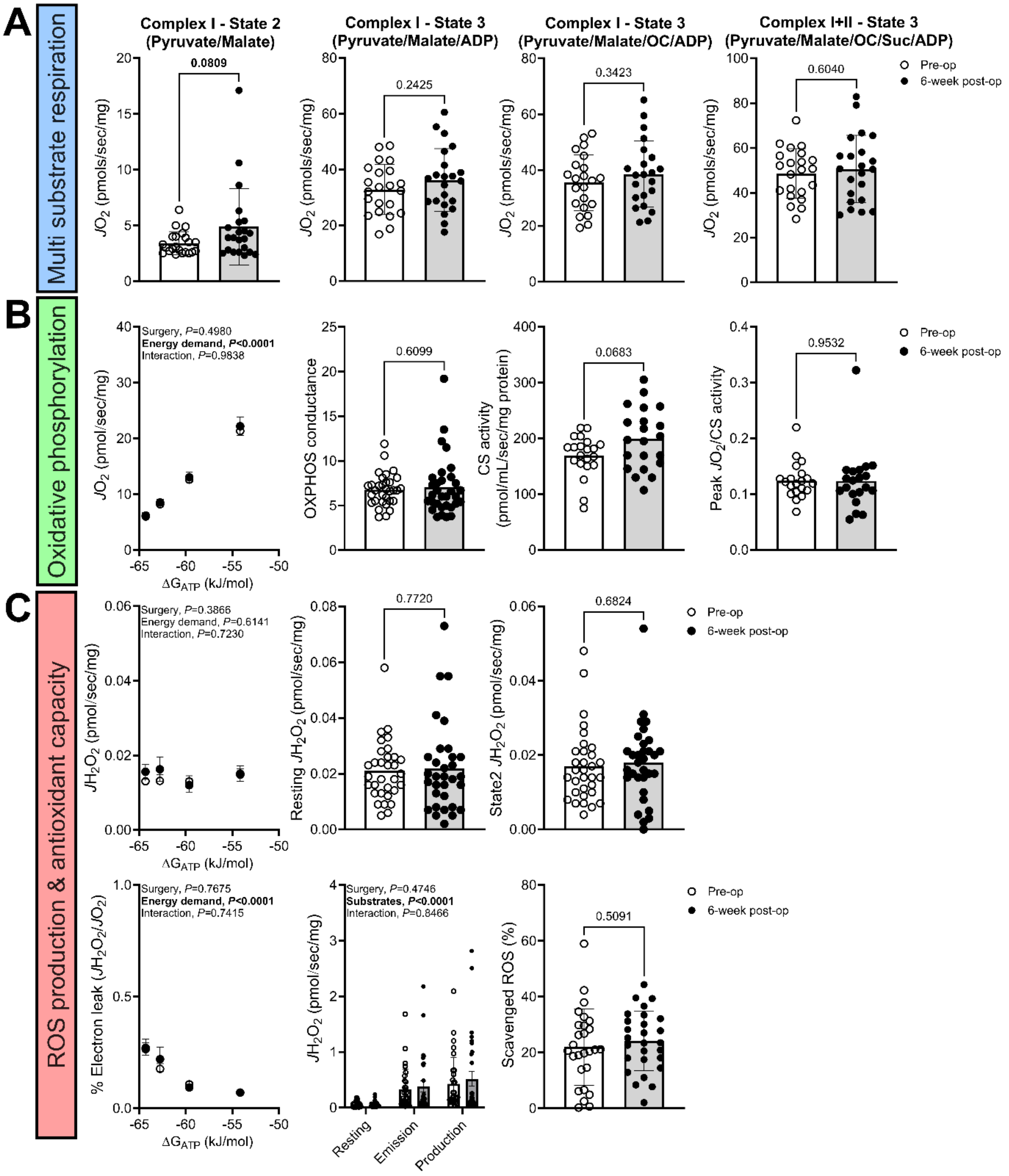

Supplemental Figure 1. Hemodialysis access creation tends to increase mitochondrial content six weeks postoperatively. (A) State 2 respiration supported by pyruvate and malate; adenosine diphosphate (ADP)-stimulated state 3 mitochondrial respiration under various substrate conditions: pyruvate and malate; pyruvate, malate, and octanoylcarnitine; and pyruvate, malate, octanoylcarnitine, and succinate (B) The

rate of oxygen consumption (JO_2) measured across varying energy demands, simulating a stress test from near-resting conditions to maximum contractions, citrate synthase (CS) activity, and maximum JO_2 normalized by CS activity, comparing values pre- and postoperatively. (C) The rate of hydrogen peroxide production (JH_2O_2) assessed under identical substrate conditions used for JO_2 measurements, along with the associated electron leak (JH_2O_2/JO_2) at both resting and state 2 JO_2 pre- and postoperatively. The lower right two panels depict JH_2O_2 at resting, along with emissions and production, and the calculated scavenging of reactive oxygen species (ROS). Data were analyzed using either a paired *t*-test or a mixed-effects analysis with Šídák's post hoc testing, and values are presented as means ± SD (N=24-34).

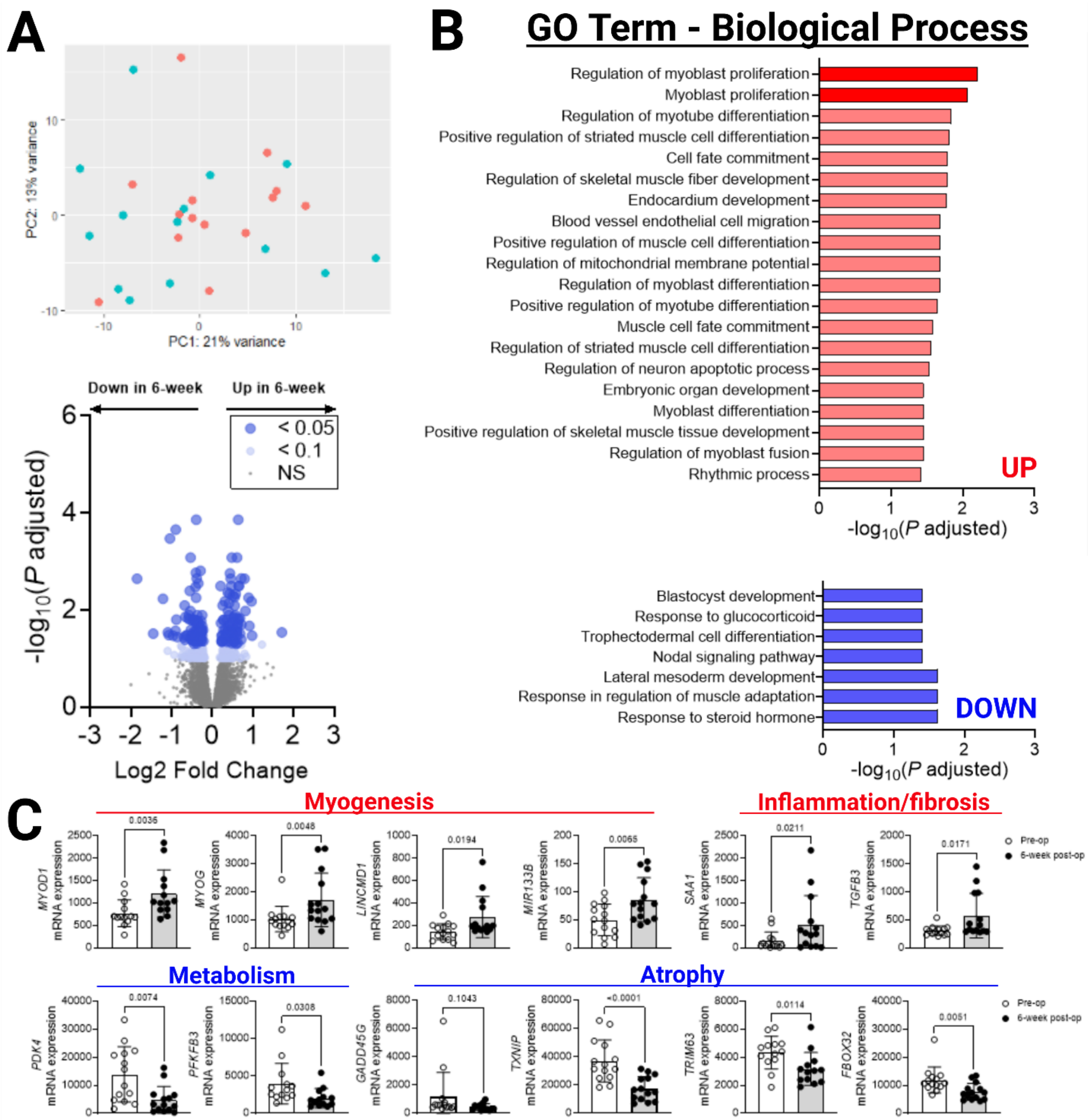

Supplemental figure 2. Bulk RNA sequencing analysis of the brachioradialis muscle. (A) principal component analysis of RNAseq results and volcano plot displaying differentially expressed genes, highlighting both up- and down-regulated genes. (B) Gene set enrichment analysis illustrating pathways significantly enriched among up- and down-regulated genes. (C) Representative mRNA expression levels of key genes that were significantly up- or down-regulated at pre-operation and six weeks post-operation (N=14).

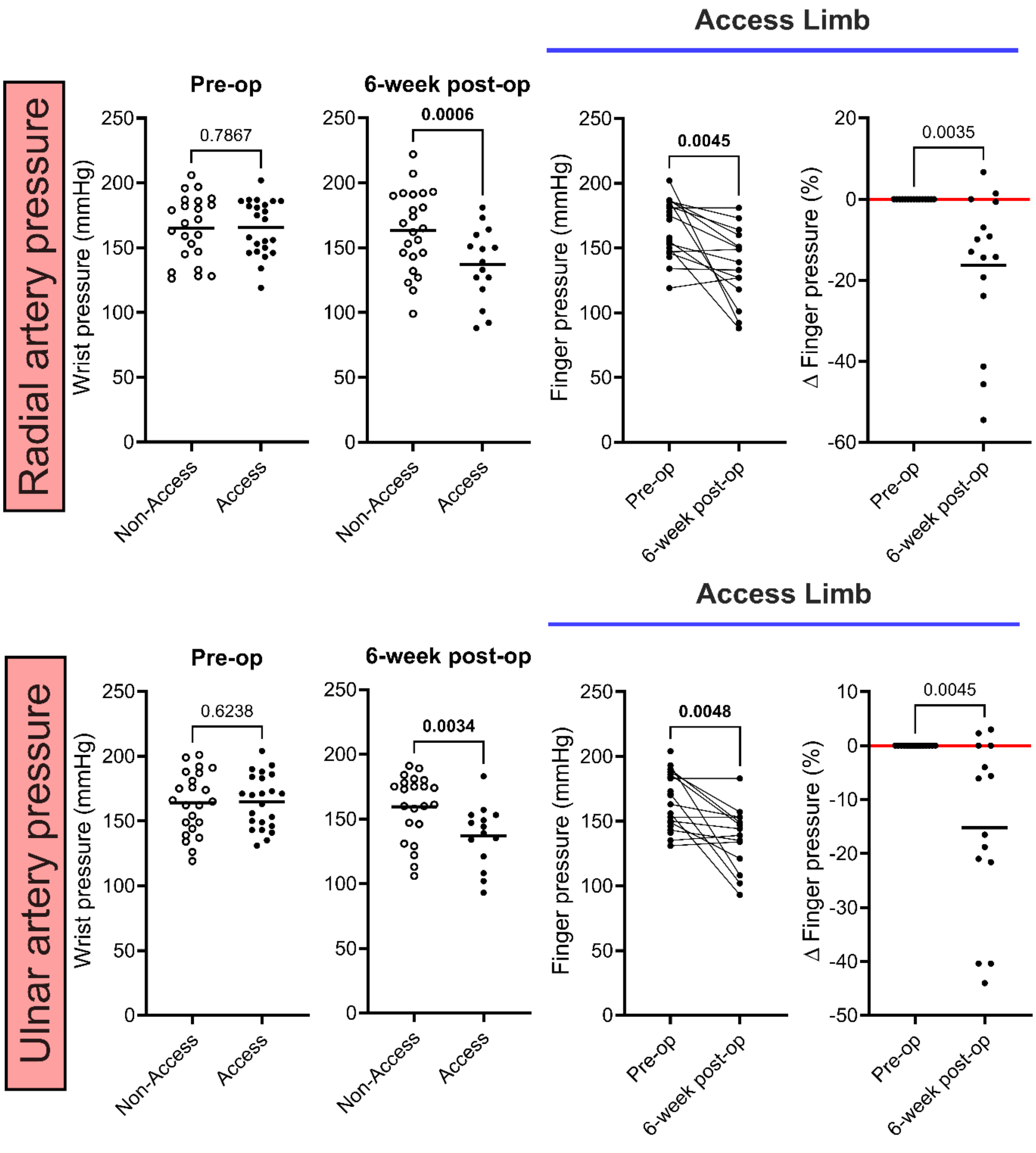

Supplemental figure 3. Subgroup analysis of wrist blood pressure. Wrist pressure comparison between non-access and access limbs at pre-operation and six weeks post-operation for radial artery (upper left two panels) and ulnar artery (lower left two panels) and comparison of raw values and percentage delta changes in wrist pressure for the access limb from pre-operation to six weeks post-operation for radial artery (upper right two panels) and ulnar artery (lower right two panels) (N=23).

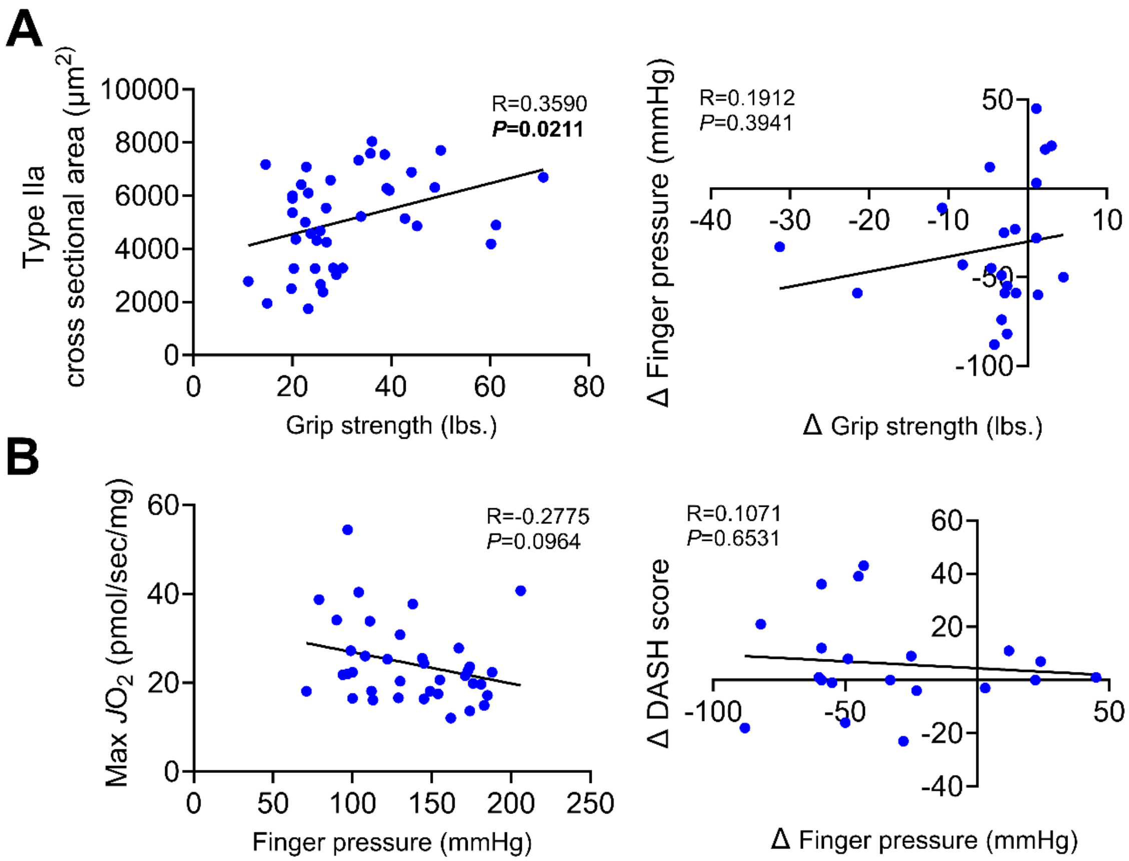

Supplemental Figure 4. Relationship between observational outcomes. (A) Pearson correlations between grip strength and variables of mean cross-sectional area of total and type IIa myofibers and postoperative delta values between grip strength and finger pressure (N=22-41). (B) Pearson correlations between finger pressure and variables of maximum JO_2 and postoperative delta values between finger pressure and DASH score (N=20-62).

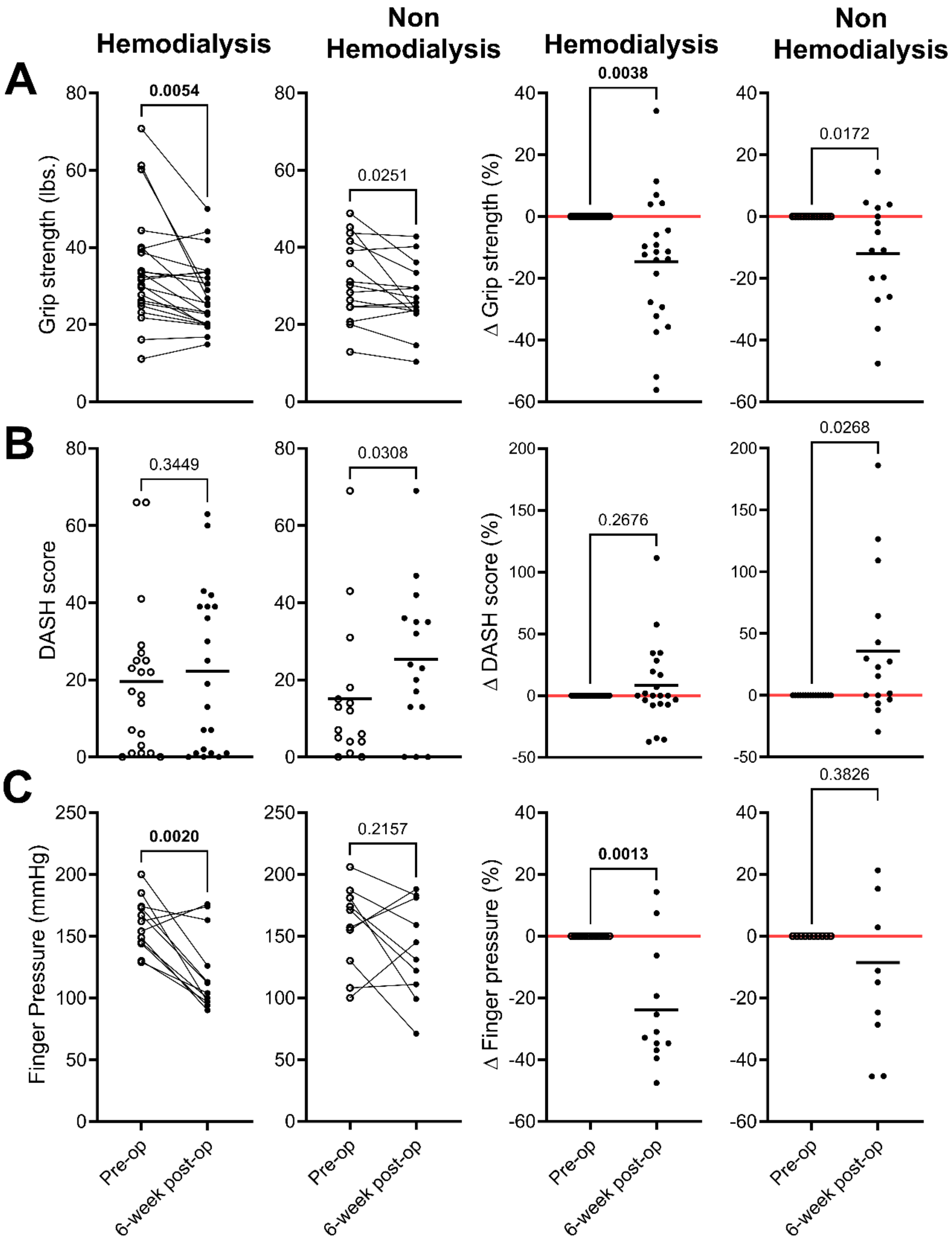

Supplemental Figure 5. Subgroup analysis of ARHD based on hemodialysis status. (A) Grip strength comparison of raw values (left two panels) and percentage delta changes (right two panels) in grip strength for the access limb from pre-operation to six weeks post-operation. (B) DASH score comparison of raw values (left two panels) and

percentage delta changes (right two panels) in DASH score for the access limb from pre-operation to six weeks post-operation. (C) Finger pressure comparison of raw values (left two panels) and percentage delta changes (right two panels) in finger pressure for the access limb from pre-operation to six weeks post-operation. (N=11-22).

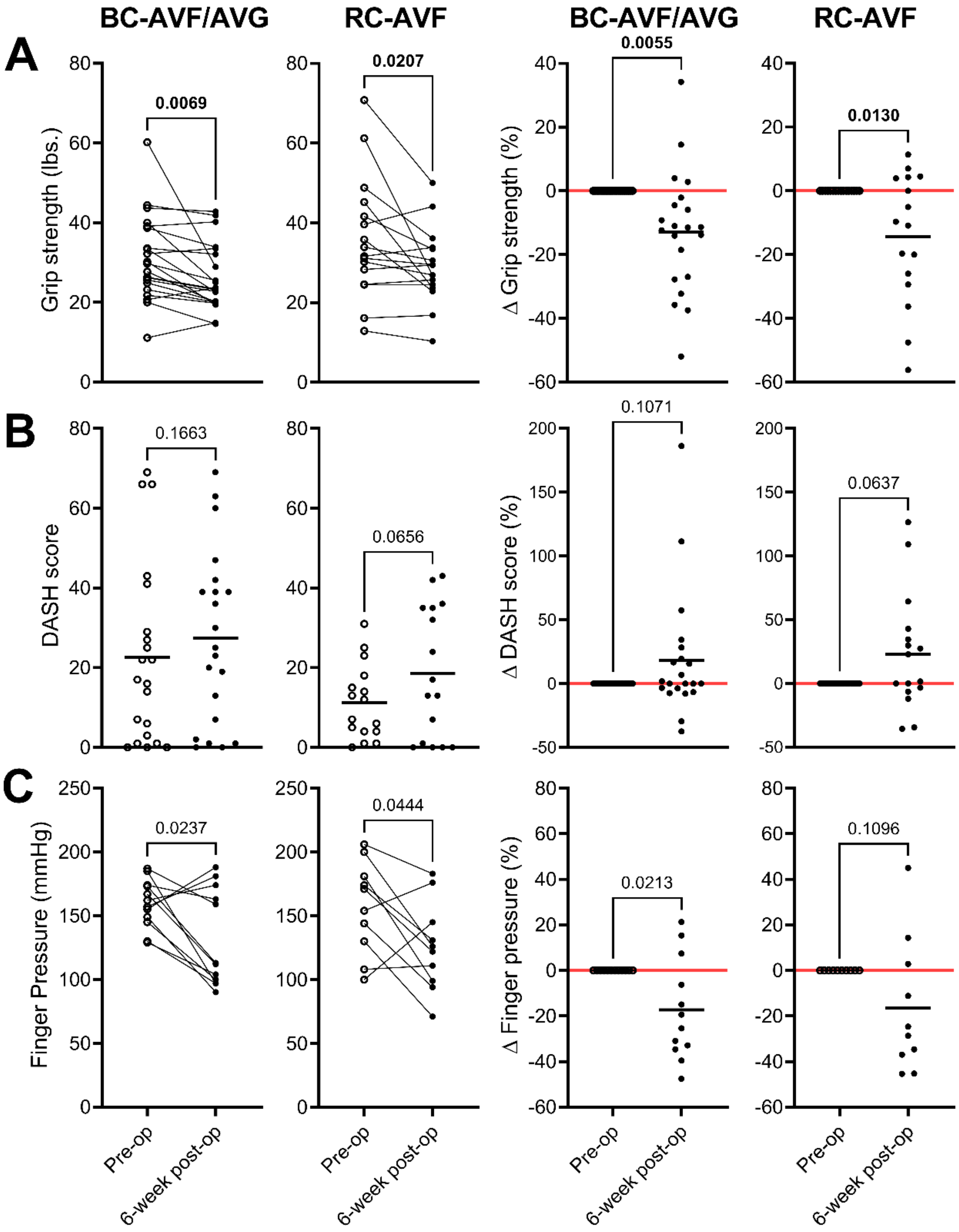

Supplemental Figure 6. Subgroup analysis of ARHD based on access site. (A) Grip strength comparison of raw values (left two panels) and percentage delta changes (right two panels) in grip strength for the access limb from pre-operation to six weeks post-operation. (B) DASH score comparison of raw values (left two panels) and percentage delta changes (right two panels) in DASH score for the access limb from pre-operation to six weeks post-operation. (C) Finger pressure comparison of raw values (left two panels)

and percentage delta changes (right two panels) in finger pressure for the access limb from pre-operation to six weeks post-operation. (N=10-21).

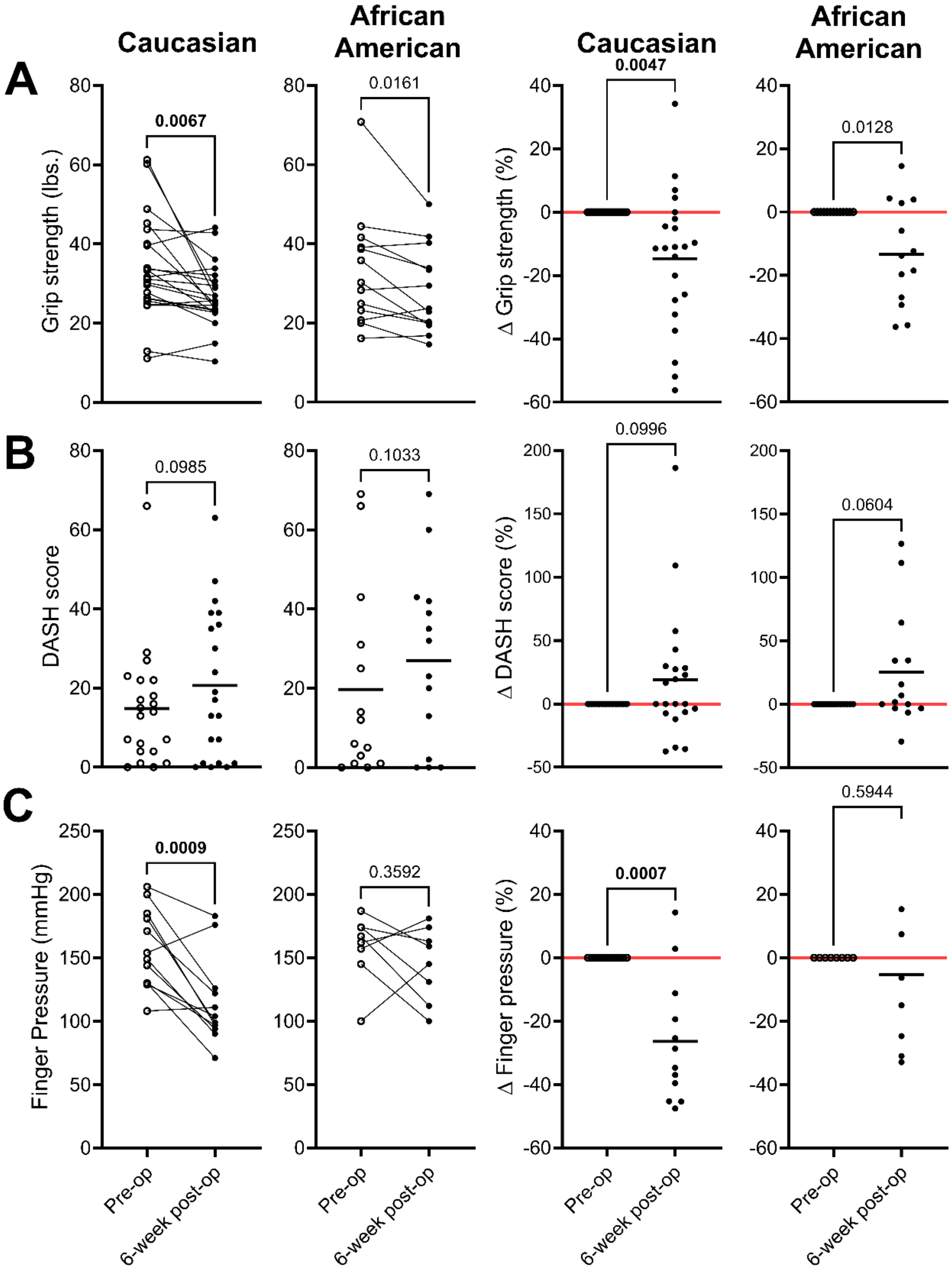

Supplemental Figure 7. Subgroup analysis of ARHD based on race. (A) Grip strength comparison of raw values (left two panels) and percentage delta changes (right two panels) in grip strength for the access limb from pre-operation to six weeks post-operation. (B) DASH score comparison of raw values (left two panels) and percentage delta changes

(right two panels) in DASH score for the access limb from pre-operation to six weeks post-operation. (C) Finger pressure comparison of raw values (left two panels) and percentage delta changes (right two panels) in finger pressure for the access limb from pre-operation to six weeks post-operation. (N=8-22).

Screening Questionnaire

Chronic Kidney Disease (Stage IV/V; estimated glomerular filtration rate < 30 ml/min/1.73m2) and/or End-stage Kidney Disease patients:

Inclusion Criteria:

1. Adults aged 18 to no upper age boundary limit
2. Planned single-stage, radial and brachial artery inflow based AVF placement or brachio-axillary bridge/ axillary-to-axillary upper arm loop graft
3. No history of previous dialysis access procedures in the study limb (but can have access history in non-study contralateral limb)
4. No history of pre-existing neuromotor disability of the study limb (i.e., known stroke with residual neuromotor deficit in the extremity receiving the access)
5. Patients should be either already on chronic in-center (or home-based) outpatient hemodialysis or anticipated to initiate hemodialysis within 3 months of study enrollment
6. Not pregnant (female participants of childbearing potential)
7. Life-expectancy > 9 months
8. Willingness to participate with the study team and comply with research protocol

Exclusion Criteria:

1. Subjects < 18 years of age
2. History of bilateral upper extremity dialysis-access procedure(s)
3. Planned 2-stage AVF placement
4. History of significant neuromotor disability involving the study limb
5. Not on hemodialysis and not anticipated to need hemodialysis within 3 months of study enrollment
6. Pregnant women
7. Life expectancy < 9 months and/or on hospice/palliative care
8. Unwilling to participate with the study team and comply with the research protocol

THE DASH

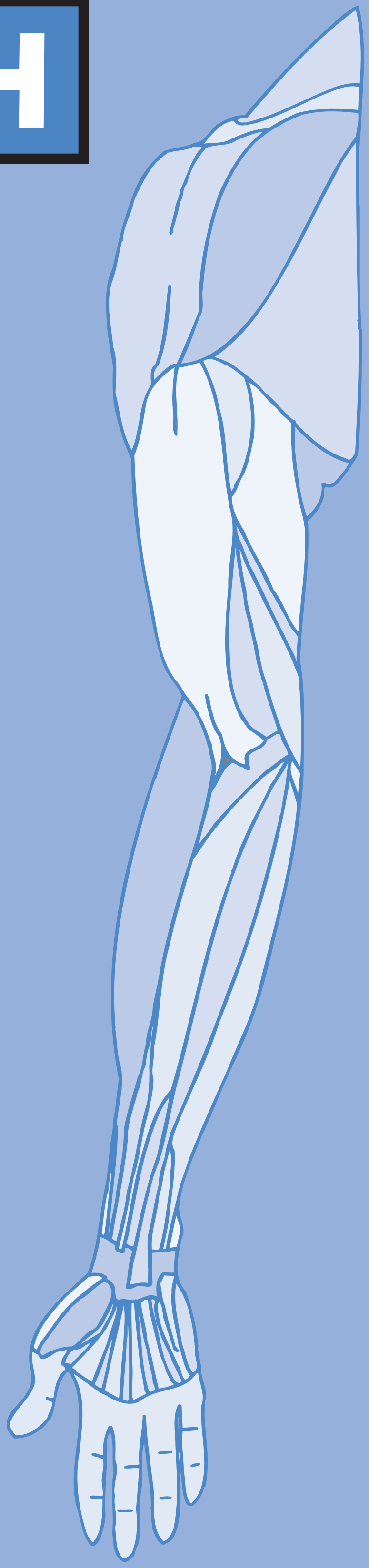

INSTRUCTIONS

This questionnaire asks about your symptoms as well as your ability to perform certain activities.

Please answer *every question*, based on your condition in the last week, by circling the appropriate number.

If you did not have the opportunity to perform an activity in the past week, please make your *best estimate* on which response would be the most accurate.

It doesn't matter which hand or arm you use to perform the activity; please answer based on your ability regardless of how you perform the task.

DISABILITIES OF THE ARM, SHOULDER AND HAND

Please rate your ability to do the following activities in the last week by circling the number below the appropriate response.

	NO DIFFICULTY	MILD DIFFICULTY	MODERATE DIFFICULTY	SEVERE DIFFICULTY	UNABLE
1. Open a tight or new jar.	1	2	3	4	5
2. Write.	1	2	3	4	5
3. Turn a key.	1	2	3	4	5
4. Prepare a meal.	1	2	3	4	5
5. Push open a heavy door.	1	2	3	4	5
6. Place an object on a shelf above your head.	1	2	3	4	5
7. Do heavy household chores (e.g., wash walls, wash floors).	1	2	3	4	5
8. Garden or do yard work.	1	2	3	4	5
9. Make a bed.	1	2	3	4	5
10. Carry a shopping bag or briefcase.	1	2	3	4	5
11. Carry a heavy object (over 10 lbs).	1	2	3	4	5
12. Change a lightbulb overhead.	1	2	3	4	5
13. Wash or blow dry your hair.	1	2	3	4	5
14. Wash your back.	1	2	3	4	5
15. Put on a pullover sweater.	1	2	3	4	5
16. Use a knife to cut food.	1	2	3	4	5
17. Recreational activities which require little effort (e.g., cardplaying, knitting, etc.).	1	2	3	4	5
18. Recreational activities in which you take some force or impact through your arm, shoulder or hand (e.g., golf, hammering, tennis, etc.).	1	2	3	4	5
19. Recreational activities in which you move your arm freely (e.g., playing frisbee, badminton, etc.).	1	2	3	4	5
20. Manage transportation needs (getting from one place to another).	1	2	3	4	5
21. Sexual activities.	1	2	3	4	5

DISABILITIES OF THE ARM, SHOULDER AND HAND

	NOT AT ALL	SLIGHTLY	MODERATELY	QUITE A BIT	EXTREMELY
22. During the past week, *to what extent* has your arm, shoulder or hand problem interfered with your normal social activities with family, friends, neighbours or groups? *(circle number)*	1	2	3	4	5

	NOT LIMITED AT ALL	SLIGHTLY LIMITED	MODERATELY LIMITED	VERY LIMITED	UNABLE
23. During the past week, were you limited in your work or other regular daily activities as a result of your arm, shoulder or hand problem? *(circle number)*	1	2	3	4	5

Please rate the severity of the following symptoms in the last week. *(circle number)*

	NONE	MILD	MODERATE	SEVERE	EXTREME
24. Arm, shoulder or hand pain.	1	2	3	4	5
25. Arm, shoulder or hand pain when you performed any specific activity.	1	2	3	4	5
26. Tingling (pins and needles) in your arm, shoulder or hand.	1	2	3	4	5
27. Weakness in your arm, shoulder or hand.	1	2	3	4	5
28. Stiffness in your arm, shoulder or hand.	1	2	3	4	5

	NO DIFFICULTY	MILD DIFFICULTY	MODERATE DIFFICULTY	SEVERE DIFFICULTY	SO MUCH DIFFICULTY THAT I CAN'T SLEEP
29. During the past week, how much difficulty have you had sleeping because of the pain in your arm, shoulder or hand? *(circle number)*	1	2	3	4	5

	STRONGLY DISAGREE	DISAGREE	NEITHER AGREE NOR DISAGREE	AGREE	STRONGLY AGREE
30. I feel less capable, less confident or less useful because of my arm, shoulder or hand problem. *(circle number)*	1	2	3	4	5

DASH DISABILITY/SYMPTOM SCORE = $\left[\left(\frac{\text{sum of n responses}}{n}\right) - 1\right] \times 25$, where n is equal to the number of completed responses.

A DASH score may <u>not</u> be calculated if there are greater than 3 missing items.

DISABILITIES OF THE ARM, SHOULDER AND HAND

WORK MODULE (OPTIONAL)

The following questions ask about the impact of your arm, shoulder or hand problem on your ability to work (including home-making if that is your main work role).

Please indicate what your job/work is:________________________________

❒ I do not work. (You may skip this section.)

Please circle the number that best describes your physical ability in the past week. Did you have any difficulty:

	NO DIFFICULTY	MILD DIFFICULTY	MODERATE DIFFICULTY	SEVERE DIFFICULTY	UNABLE
1. using your usual technique for your work?	1	2	3	4	5
2. doing your usual work because of arm, shoulder or hand pain?	1	2	3	4	5
3. doing your work as well as you would like?	1	2	3	4	5
4. spending your usual amount of time doing your work?	1	2	3	4	5

SPORTS/PERFORMING ARTS MODULE (OPTIONAL)

The following questions relate to the impact of your arm, shoulder or hand problem on playing *your musical instrument or sport or both*. If you play more than one sport or instrument (or play both), please answer with respect to that activity which is most important to you.

Please indicate the sport or instrument which is most important to you:________________________________

❑ I do not play a sport or an instrument. (You may skip this section.)

Please circle the number that best describes your physical ability in the past week. Did you have any difficulty:

	NO DIFFICULTY	MILD DIFFICULTY	MODERATE DIFFICULTY	SEVERE DIFFICULTY	UNABLE
1. using your usual technique for playing your instrument or sport?	1	2	3	4	5
2. playing your musical instrument or sport because of arm, shoulder or hand pain?	1	2	3	4	5
3. playing your musical instrument or sport as well as you would like?	1	2	3	4	5
4. spending your usual amount of time practising or playing your instrument or sport?	1	2	3	4	5

SCORING THE OPTIONAL MODULES: Add up assigned values for each response; divide by 4 (number of items); subtract 1; multiply by 25.
An optional module score may not be calculated if there are any missing items.

Institute for Work & Health | Research Excellence Advancing Employee Health

© INSTITUTE FOR WORK & HEALTH 2006. ALL RIGHTS RESERVED.
